# Acrylamide-dT: a polymerisable nucleoside for DNA incorporation†

**DOI:** 10.1039/c9ra07570d

**Published:** 2019-10-03

**Authors:** Francia Allabush, Paula M. Mendes, James H. R. Tucker

**Affiliations:** School of Chemistry, University of Birmingham Edgbaston Birmingham B15 2TT UK j.tucker@bham.ac.uk; School of Chemical Engineering, University of Birmingham Edgbaston Birmingham B15 2TT UK p.m.mendes@bham.ac.uk

## Abstract

The synthesis of a novel modified nucleoside phosphoramidite, Acrylamide-dT-CE phosphoramidite, obtained in three steps from commercially available starting materials, is reported. It was readily incorporated into thrombin binding aptamer (TBA) sequences using automated solid-phase synthesis under ultra-mild conditions, with the modification shown not to adversely affect duplex stability, G-quadruplex structure, or thrombin binding. The reaction and integration of the modified strands with acrylamide polymers was evidenced by gel electrophoresis. The Acrylamide-dT functional handle promises to be an ideal synthon for preparing DNA–polymer hybrids for use in various macromolecular materials applications.

## Abstract 2

Nucleoside derivative Acrylamide-dT can be readily synthesised and incorporated multiple times into DNA sequences at any position *via* automated synthesis.

## Graphical abstract

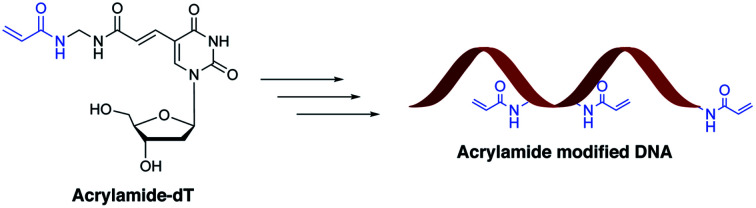

## Introduction

**Fig. 1:**
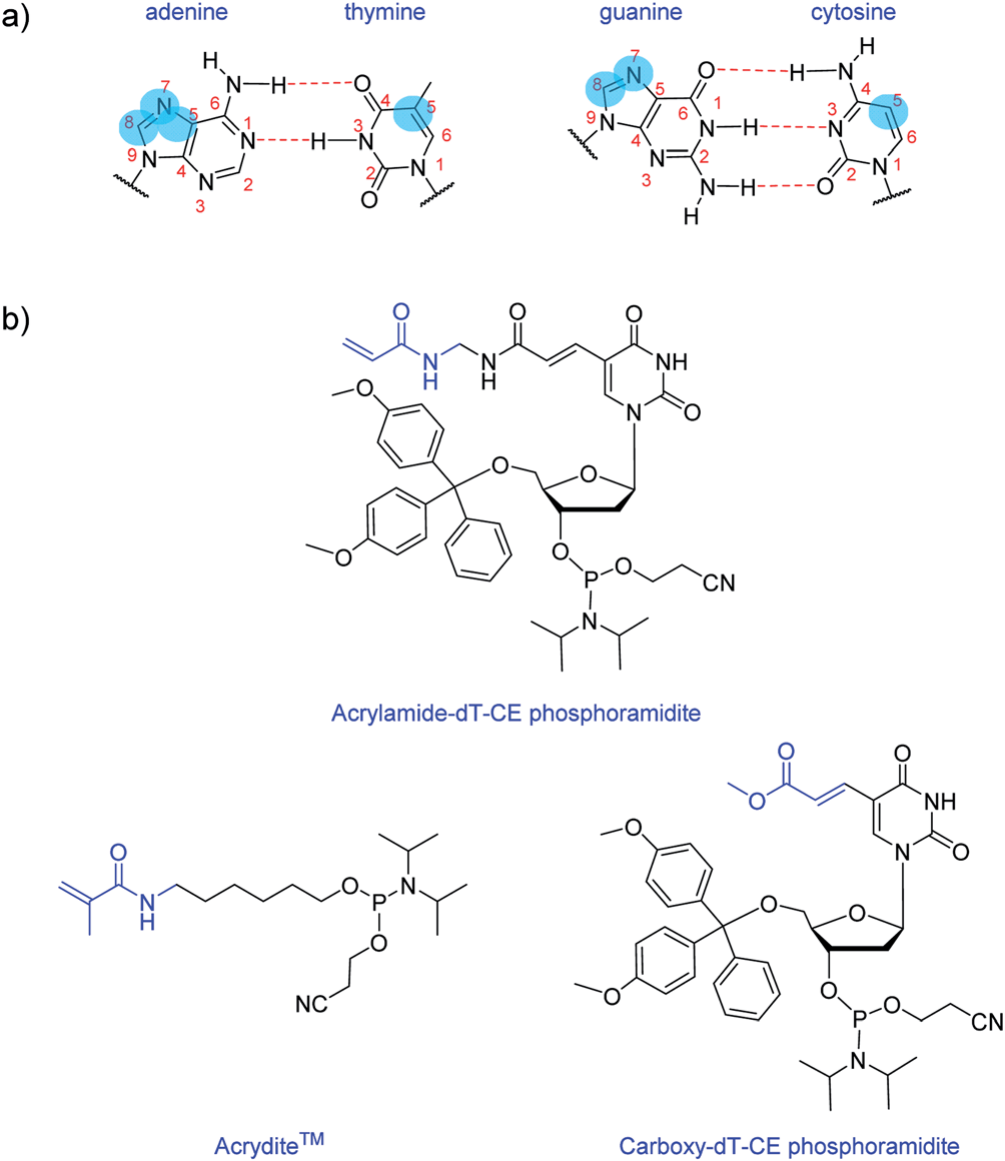
Structures of: (a) canonical H-bonding DNA base pairs with typical modification positions highlighted in blue; (b), Acrylamide-dT-CE phosphoramidite, Acrydite™ and Carboxy-dT-CE phosphoramidite.

Nucleobase modifications are commonplace for enhancing the properties of oligonucleotides for therapeutic and diagnostic applications. Such modifications are typically employed for improving duplex stability,^1^ increasing binding affinity and selectivity towards certain targets^2,3^ or introducing detection elements such as fluorescent^4^ or redox-active^5^ groups. The C5 site of pyrimidines and the N7/C8 sites of purines are usually selected for modification as they are more synthetically accessible than other sites and do not interfere with base pairing interactions (Fig. 1a).^6^ And largely too for synthetic reasons, the most common nucleobase for functionalisation is thymine (or uracil), as fewer protective steps are required. Functional reactive handles sited at the C5 position of thymine/uracil are typically amino,^7^ alkyne,^8^ carboxylate,^9,10^ halogenated,^11^ or thiol^12^ groups. Once inserted into DNA, these handles may be used to conjugate the oligonucleotide (*i.e.* using post-DNA synthesis) to various motifs *via* amide coupling,^13^ palladium catalysed coupling,^14^ or alkyne–azide click chemistry.^15^

Here, we introduce an acrylamide-containing group at the C5 position of thymine to create the novel nucleoside derivative Acrylamide-dT in the form of its phosphoramidite (Fig. 1b). This new handle is ideal for reacting with nucleophiles such as thiols *via* the Michael addition reaction, or for forming polymers *via* free radical polymerisation chemistry. Currently for such purposes, oligonucleotides can be modified with the commercially available phosphoramidite Acrydite™ (Fig. 1b).^16^ For example, Jie and coworkers used this acrylamide reagent to integrate fluorescent DNA into hydrogels, whereas others have incorporated it into aptameric systems for molecular imprinting applications.^9,10,17,18^ However, the placement of Acrydite™ is limited to the 5′ end of the oligonucleotide. We reasoned that a synthon such as Acrylamide-dT would allow placement of acrylamide moieties not only internally, but also at multiple positions within a strand.

Multiple incorporations of polymerisable groups into DNA have previously been performed using the commercially available Carboxy-dT-CE monomer (Fig. 1b), with the ester moiety reported as being converted to an amide upon oligonucleotide deprotection with ammonia.^9^ The work by Turner *et al.* on DNA-incorporated molecularly imprinted nanoparticles highlights that multiple anchorage points of the DNA strand to the polymer network is advantageous for target binding.^9,10^ A structural comparison of this Carboxy-dT-CE monomer to Acrylamide-dT suggests heightened reactivity for the latter through its primary alkene which is less sterically hindered. Herein we report the ready incorporation of Acrylamide-dT into DNA sequences at both internal and end positions and also show that these strands can undergo polymerisation with acrylamide/bisacrylamide monomers in gel electrophoresis experiments.

## Results and discussion

### Synthesis of Acrylamide-dT and its phosphoramidite

**Scheme 1:**
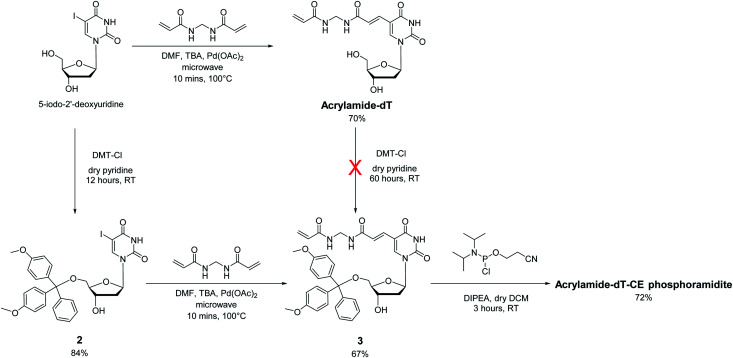
Synthesis of Acrylamide-dT and its derivatives.

In approaching the synthesis of the target Acrylamide-dT and its corresponding phosphoramidite, our first strategy involved replacing the iodine at C5 of commercially available 5-iodo-2′-deoxyuridine with an acrylamide group *via* the Heck reaction with *N*,*N*′-methylenebisacrylamide (Scheme 1). A microwave assisted procedure adapted from Fujimoto^19^ was followed to couple the two entities together to form Acrylamide-dT in good yield (70%). Excess *N*,*N*′-methylenebisacrylamide was used to minimise coupling to both sides of the molecule. The microwave reaction was monitored by thin layer chromatography, which took approximately 10 minutes to complete. The product was isolated by precipitation from cold chloroform, and washed with cold chloroform to remove excess *N*,*N*′-methylenebisacrylamide. The next step was to perform tritylation and phosphitylation to allow incorporation of the target molecule into DNA sequences using automated synthesis. Unfortunately, tritylation of the 5′ hydroxyl position of Acrylamide-dT with a dimethoxytrityl group proved difficult, resulting in extremely low yields of compound 3 (<5%) even under prolonged reaction conditions (3 days). An alternative route involving a change in order was therefore devised in which tritylation was performed first on 5-iodo-2′-deoxyuridine to give compound 2 (ref. 20) prior to the palladium catalysed addition of *N*,*N*′-methylenebisacrylamide. This route proved successful with identical microwave conditions applied to produce compound 3 in good yield. The enhanced hydrophobicity of compound 3 led to its isolation by column chromatography using DCM/methanol rather than precipitation from chloroform. The remaining 3′ alcohol of compound 3 was then phosphitylated to obtain the target Acrylamide-dT-CE phosphoramidite in 72% yield (Scheme 1).

### Incorporation of Acrylamide-dT into DNA

Preliminary ^1^H, ^13^C, and 2D NMR, and mass spectrometry experiments on Acrylamide-dT showed that the phosphoramidite would not be able to withstand standard oligonucleotide deprotection conditions used in DNA synthesis as ammonia was found to add to the terminal alkene to form a primary amine (see ESI†). However no such problems were found using the ultramild deprotection conditions of methanolic potassium carbonate. Therefore these conditions were chosen for automated oligonucleotide synthesis. Given the ongoing interest in functionalising aptameric DNA sequences with polymerisable groups,^9,10,17,18^ and our recent work on such systems,^21^ it was decided to incorporate Acrylamide-dT into the thrombin binding aptamer (TBA). Different positions in the TBA sequence were chosen, including internal T sites and end-functionalisation, as shown in Table 1. Those T sites in the central TGT loop known not to interact with the thrombin protein were selected for replacement with Acrylamide-dT.^22^ DNA synthesis proceeded very smoothly with up to three incorporations achieved successfully, and high average stepwise yields of >90% for each coupling. Each strand was purified by reversed-phase HPLC (RP-HPLC) and characterised by analytical RP-HPLC and ESMS (see ESI†). The strands were found to be stable, with no appreciable degradation after storing samples under standard conditions (ultrapure water at −20 °C) over several months.

**Table 1:** Unmodified and Acrylamide-dT modified DNA sequences (denoted by **X**)

Oligo	Sequence (**X** = Acrylamide-dT unit)
TBA	5′-GGTTGGTGTGGTTGG-3′
Acryl-endT	5′-**X**GGTTGGTGTGGTTGG-3′
Acryl-T7/T9	5′-GGTTGG**X**G**X**GGTTGG-3′
Acryl-endT/T7/T9	5′-**X**GGTTGG**X**G**X**GGTTGG-3′

### Structure and binding properties of Acrylamide-dT modified DNA

**Fig. 2:**
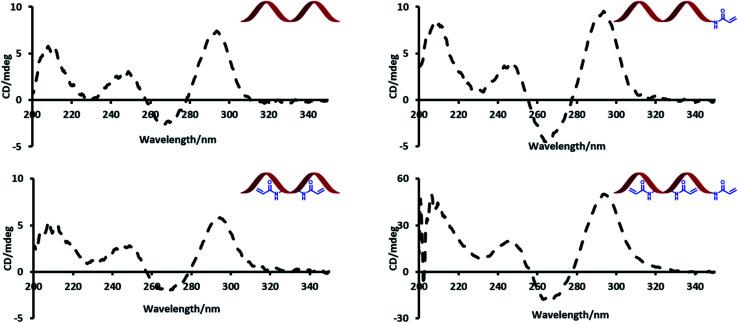
CD spectra of unmodified (TBA) and Acrylamide-dT modified DNA strands.

Circular dichroism (CD) spectroscopy was used to probe the effect of introducing acrylamide groups on the structure of the modified aptamer strands (Fig. 2). In the presence of potassium, TBA has a characteristic CD maximum at *ca.* 295 nm and a CD minimum at *ca.* 265 nm, which is indicative of a G-quadruplex conformation.^23^ The data showed no significant difference in peak positions of the CD signals of the modified strands compared to the unmodified TBA strand. The strong peaks at *ca.* 295 nm highlight this effect and indicate that all modified strands can similarly adopt a G-quadruplex conformation.

Thermal melting (*T*_m_) experiments were performed to assess whether the inclusion of acrylamide groups had any effect on the stability of the duplexes formed with the 15-mer complementary strand (Table 2). The average *T*_m_ values of the modified strands were similar if not slightly higher than the unmodified version. This suggests that the acrylamide groups enhance the stability of the duplex to some extent, possibly since the acrylamide moiety has sites for additional hydrogen bonding interactions.

**Table 2:** *T*_m_ values for complementary duplexes formed with unmodified (TBA) and Acrylamide-dT modified DNA

Oligo	Average *T*_m_/°C	Std. dev./°C
TBA	62.7	2.9
Acryl-endT	65.8	0.7
Acryl-T7/T9	64.2	0.5
Acryl-endT/T7/T9	66.7	0.2

Gel electromobility shift assays were then used to demonstrate affinity of the Acrylamide-dT modified oligonucleotides to the thrombin protein (see ESI†). The images revealed near complete binding to thrombin for all modified stands at a ratio of 1 : 1 DNA : thrombin. Furthermore, the intensities of the bands were similar to those observed with unmodified TBA, indicating similar thrombin binding affinities, with the dissociation constant (*K*_d_) ratio for TBA and Acryl-endT estimated from relative band intensities as: *K*_d_^Acryl-endT^/*K*_d_^TBA^ = 1.2.

### Reaction of Acrylamide-dT modified DNA with acrylamide gels

**Fig. 3:**
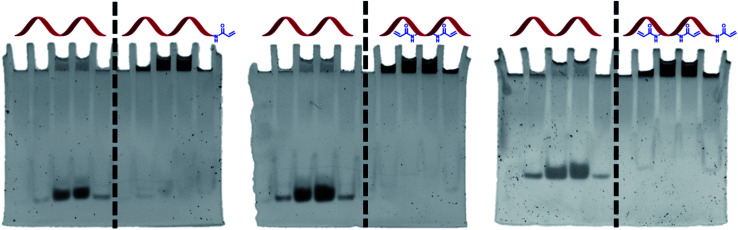
Three polyacrylamide gel experiments involving unmodified (TBA, left of vertical dashed line) and Acrylamide-dT modified DNA (right of vertical dashed line). In each gel, samples of each strand were loaded into two adjacent wells.

An important aspect of the work was to check whether DNA functionalisation would not adversely affect the ability of the acrylamide groups to polymerise. A polyacrylamide gel experiment was therefore designed to test this in the presence of other acrylamide monomers. A mixture of acrylamide, bisacrylamide, ammonium persulphate, TEMED, and DNA (unmodified or Acrylamide-dT modified) was loaded into wells of a polyacrylamide gel and allowed to set. It was anticipated that if the acrylamide group on the strands could react with the gel, the strands would become covalently bound and immobilised. Conversely, unmodified TBA would move down the gel in the normal way as it does not possess the acrylamide functionality. Once run, the gels were stained and visualised to determine the position of the strands (Fig. 3). As expected, each Acrylamide-dT containing DNA strand remained in the wells at the top of the gel. The absence of any band intensity lower down each gel suggests a near-quantitative reaction under these conditions. In contrast, samples of unmodified TBA moved down the gel along with the current, with there being no indication of any reaction with acrylamide monomers, for example *via* amine-containing nucleobases. This result clearly shows the ability of the incorporated acrylamide groups to react with other acrylamide monomers to anchor the DNA within the polymer network. As expected, running gels of the three modified strands in the absence of acrylamide, bisacrylamide and radical initiators resulted in no retardation of the bands (see ESI†).

## Conclusion

In conclusion, a new polymerisable nucleosidic monomer, Acrylamide-dT, and its associated phosphoramidite has been synthesised, with the latter made in three steps from commercially available starting materials. Under ultramild DNA synthesis conditions, it can be readily incorporated intact into aptamer sequences, both internally and externally (*i.e.* at strand ends) and in multiple positions, thus expanding the repertoire for DNA modification chemistry. The modifications were found to have no adverse effect on the structures or thrombin binding properties of the DNA strands, as evidenced by CD, *T*_m_ and gel experiments. Furthermore, the acrylamide groups within these strands were shown to react with other acrylamide monomers under gel electrophoresis conditions to form acrylamide polymers. We expect Acrylamide-dT to be a useful synthon in the future for constructing new DNA–polymer hybrids for various applications such as stimuli-responsive hydrogels or molecular imprinting. Although this methodology has currently been tested only on thymine/uracil sites, given the ease of synthesis, it should be possible to similarly attach acrylamide groups to other nucleobases to broaden the applicability further.

## Experimental

### Materials and methods

Reagents and solvents were purchased from commercial suppliers and used without further purification, unless otherwise stated. 5′-*O*-(4,4′-dimethoxytrityl)-5-iodo-2′-deoxyuridine 2 was synthesised following an established procedure.^20^ Column chromatography was carried out using open columns packed with Merck grade 60 silica gel topped with 0.5 cm of sand. TLC analysis was performed on Merck silica gel 60 silica sheets. ^1^H, ^13^C, and ^31^P NMR spectra were obtained on Bruker AVIII300 or AVIII400 spectrometers. Chemical shifts (*δ*) are given in ppm and are relative to the residual solvent peak. Electrospray mass (ESI-MS) spectra were measured by either Waters micromass LCT electrospray time-of-flight (ES-TOF), Waters Xevo G2-XS, or Synapt G2S mass spectrometers. Milli-Q water purified with a Millipore Elix-Gradient A10 system (resistivity > 18 μΩ cm, TOC ≤ 5 ppb, Millipore, France) was used for DNA sample preparation.

### Synthesis of Acrylamide-dT

5-Iodo-2′-deoxyuridine (1.00 g, 2.82 mmol) and palladium acetate (60 mg, 0.28 mmol) were suspended in DMF (3 ml) in a 10 ml microwave tube equipped with a small magnetic stirring bar. Tributylamine (0.67 ml, 2.82 mmol) and *N*,*N*′-methylenebisacrylamide (1.09 g, 7.06 mmol), were then added. The suspension was stirred and degassed with argon for 10 minutes. The tube was then tightly sealed, and the mixture irradiated in a microwave for 10 minutes at 100 °C. After the irradiation period, the reaction vessel was cooled to room temperature before opening. The reaction was then filtered through Celite®. The product was isolated by precipitation from cold chloroform, washing with cold chloroform, to yield an off white solid (748 mg, 70%). ^1^H NMR (400 MHz, (CD_3_)_2_SO) *δ* 11.56 (s, 1H), 8.69 (t, *J* = 5.9 Hz, 2H), 8.32 (s, 1H), 7.18 (d, *J* = 15.6 Hz, 1H), 6.96 (d, *J* = 15.6 Hz, 1H), 6.30–6.21 (m, 1H), 6.16–6.09 (m, 2H), 5.64–5.58 (m, 1H), 5.27 (d, *J* = 4.3 Hz, 1H), 5.16 (t, *J* = 5.0 Hz, 1H), 4.54 (t, *J* = 5.8 Hz, 2H), 4.29–4.23 (m, 1H), 3.82–3.78 (m, 1H), 3.70–3.55 (m, 1H), 2.18–2.13 (m, 2H). ^13^C NMR (100 MHz, (CD_3_)_2_SO) *δ* 166.0 (C=O), 164.8 (C=O), 161.8 (C=O), 149.4 (C=O), 142.4 (HC=C), 132.9 (HC=C), 131.5 (HC=C), 125.8 (H_2_C=C), 120.8 (HC=C), 108.9 (C), 87.6 (CH), 84.6 (CH), 69.9 (CH), 60.9 (CH_2_), 43.6 (CH_2_), 40.0 (CH_2_). HRMS (ES +ve) (*m*/*z*): [M + Na]^+^ calcd for C_16_H_20_N_4_O_7_Na, 403.1230; found 403.1229. IR neat (cm^−1^): 3295 (m, OH/NH), 3065 (w, CH), 1689 (s, C=O), 1650 (s, C=C). Mp: 250 °C (degradation).

### Synthesis of compound 3

Compound 2 (ref. 20) (1.01 g, 1.54 mmol) and palladium acetate (35 mg, 0.16 mmol) were suspended in DMF (3 ml) in a 10 ml microwave tube equipped with a small magnetic stirring bar. Tributylamine (0.37 ml, 1.56 mmol) and *N*,*N*′-methylenebisacrylamide (475 mg, 3.08 mmol) were then added. The suspension was stirred and degassed with argon for 10 minutes. The tube was tightly sealed and the mixture irradiated in a microwave for 10 minutes at 100 °C. After the irradiation period, the reaction vessel was cooled to room temperature before opening. The reaction was then filtered through Celite®, rinsing with DCM (50 ml), and the filtrate washed with water (50 ml). The organic phase was dried over magnesium sulphate and evaporated under vacuum. The crude product was purified by flash chromatography with an eluent of 9 : 1 DCM : 7 N ammonia in methanol. The appropriate fractions were combined and evaporated to give an off white solid (700 mg, 67%).

^1^H NMR (400 MHz, CDCl_3_) *δ* 10.87 (s, 1H), 8.14 (s, 1H), 7.86 (s, 1H), 7.39–7.37 (m, 3H), 7.29–7.25 (m, 7H), 7.20–7.14 (m, 2H), 6.84 (d, *J* = 8.7 Hz, 4H), 6.36–6.28 (m, 2H), 6.14–6.08 (m, 1H), 5.66 (d, *J* = 11.1 Hz, 1H), 4.75 (t, *J* = 6.6 Hz, 2H), 4.46 (s, 1H), 4.24–4.21 (m, 1H), 3.74 (s, 6H), 3.44–3.31 (m, 2H), 2.70–2.66 (m, 1H), 2.23–2.17 (m, 1H), 1.81 (s, 1H). ^13^C NMR (100 MHz, CDCl_3_) *δ* 168.1 (C=O), 166.8 (C=O), 162.2 (C=O), 158.8 (Ar–C), 149.6 (C=O), 144.4 (Ar–C), 142.2 (HC=C), 135.7 (Ar–C), 135.5 (Ar–C), 133.9 (HC=C), 130.3 (HC=C), 130.2 (Ar–CH), 130.0 (Ar–CH), 128.3 (H_2_C=C), 128.2 (Ar–CH), 127.2 (Ar–CH), 121.9 (HC=C), 113.5 (Ar–CH), 110.1 (C), 87.2 (C), 87.0 (CH), 86.8 (CH), 72.8 (CH), 64.0 (CH_2_), 55.4 (OCH_3_), 44.5 (CH_2_), 41.6 (CH_2_). HRMS (ES +ve) (*m*/*z*): [M + H]^+^ calcd for C_37_H_39_N_4_O_9_, 683.2717; found 683.2715. IR neat (cm^−1^): 3483 (b, OH/NH), 2921 (s, CH), 2851 (s, CH), 1607 (m, C=O).

### Synthesis of Acrylamide-dT-CE phosphoramidite

Compound 3 (771 mg, 1.13 mmol) was dried *via* azeotroping with dry DCM (3 × 10 ml). The solid was then redissolved in dry DCM (15 ml), and the solution stirred under argon. DIPEA (0.49 ml, 2.84 mmol) was added *via* syringe to the solution. Then 2-cyanoethyl *N*,*N*-diisopropylchlorophosphoramidite (0.3 ml, 1.34 mmol) was cautiously added to the mixture. The solution was left to stir under argon until reaction completion was confirmed by TLC (∼3 hours). The reaction was diluted with degassed DCM (50 ml), and the solution was washed with degassed saturated sodium bicarbonate solution (2 × 50 ml). The organic phases were collected, dried over sodium sulphate, and evaporated under vacuum. The crude product was purified by flash chromatography with an eluent of 9 : 1 DCM : 7 N ammonia in methanol. The appropriate fractions were combined and evaporated to give an off white solid (717 mg, 72%).

^1^H NMR (400 MHz, CDCl_3_) *δ* 8.65–8.59 (m, 1H), 7.79 (d, *J* = 21.1 Hz, 1H), 7.43–7.40 (m, 2H), 7.33–7.27 (m, 8H), 7.23–7.18 (m, 1H), 7.07 (dd, *J* = 15.3, 2.7 Hz, 1H), 6.87–6.83 (m, 4H), 6.44 (dd, *J* = 17.0, 1.1 Hz, 1H), 6.28–6.23 (m, 1H), 6.16–6.09 (m, 1H), 5.69 (dd, *J* = 10.4, 1.0 Hz, 1H), 4.73 (t, *J* = 6.0 Hz, 2H), 4.56–4.49 (m, 1H), 4.28–4.22 (m, 1H), 3.87–3.52 (m, 11H), 3.38–3.29 (m, 2H), 2.72–2.59 (m, 2H), 2.46 (t, *J* = 6.4 Hz, 1H), 2.28–2.17 (m, 1H), 1.18–1.08 (m, 12H). ^13^C NMR (100 MHz, CDCl_3_) *δ* 167.8 (C=O), 166.6 (C=O), 162.8 (C=O), 158.6 (Ar–C), 149.1 (C=O), 144.3 (Ar–C), 142.1 (HC=C), 135.6 (Ar–C), 135.4 (Ar–C), 133.5 (HC=C), 130.1 (HC=C), 130.0 (Ar–CH), 128.4 (H_2_C=C), 128.1 (Ar–CH), 128.0 (Ar–CH), 127.1 (Ar–CH), 122.0 (HC=C), 117.5/117.4 (CN), 113.4 (Ar–CH), 109.9/109.8 (C), 86.8 (C), 86.3–86.0 (CH), 85.7 (CH), 74.0–73.5 (CH), 63.4/63.3 (CH_2_), 58.4/58.2 (CH_2_), 55.2 (OCH_3_), 44.6 (CH_2_), 43.4–43.2 (CH), 40.4 (CH_2_), 24.6 (CH_3_), 20.4–20.2 (CH_2_). ^31^P NMR (121 MHz, CDCl_3_) *δ* 149.1, 148.6. HRMS (TOF-ES +ve) (*m*/*z*): [M]^+^ calcd for C_46_H_56_N_6_O_10_P, 883.3796; found 883.3793.

### Synthesis of unmodified and Acrylamide-dT modified DNA oligonucleotides

All oligonucleotides were synthesised using solid phase synthesis on an Applied Biosystems ABI 394 DNA/RNA synthesiser using commercially supplied DNA synthesis grade solvents and reagents. Standard phosphoramidites of Pac-dA, iPr-Pac-dG, Ac-dC, dT from Link Technologies, and Acrylamide-dT-CE phosphoramidite were used for ultramild synthesis. The phosphoramidites were dissolved in anhydrous acetonitrile to 0.1 M prior to synthesis. Strands were synthesised at a 1 μmol scale on SynBase™ CPG 1000/110 solid supports from Link Technologies. Phosphoramidites were activated with 5-ethylthio-1*H*-tetrazole (0.25 M) in acetonitrile prior to coupling and coupling times of 25 seconds were used. Then, phenoxyacetic anhydride and methylimidazole were added to cap unreacted material, and iodine (0.02 M) in THF/pyridine/water (7 : 2 : 1) was added to oxidise the phosphotriester formed. Upon sequence completion, the resins were placed in freshly prepared 1 ml solutions of potassium carbonate (0.05 M) in methanol and left overnight to cleave strands from the resin and remove protecting groups. The solutions were neutralised with acetic acid (6 μl) and the solvent was removed on a Thermo Scientific speed vac. The dried powders were redissolved in 1 ml Milli-Q water and desalted with a NAP-10 column from GE Healthcare to remove residual resin and potassium carbonate. The solutions were then concentrated to 1 ml and stored in the freezer for purification (see ESI for further details†).

### Circular dichroism spectroscopy

500 μl aqueous solutions of each DNA sample (5 μM) in KCl (10 mM) and Tris·HCl buffer (10 mM, pH 7.5) were prepared. Prior to CD analysis, the samples were heated to 95 °C for 5 minutes and cooled slowly to room temperature. CD spectra of samples were recorded on a Jasco J-810 spectropolarimeter, scanning from 350–200 nm at a rate of 100 nm min^−1^. Three accumulations were performed for each sample and the data produced an average of the three scans. A baseline correction was manually performed on each sample by subtracting the blank and offsetting results at 350 nm.

### Thermal melting experiments

100 μl aqueous solutions of each DNA sample (10 μM) and complementary DNA (10 μM) in NaCl (100 mM) and sodium phosphate buffer (10 mM, pH 7) were prepared. 10 μl of each sample was withdrawn, added to 10 μL of SsoAdvanced™ universal SYBR® green supermix from Bio-rad, and the thermal melting of the resulting solutions was performed on a M550 double beam scanning UV/visible spectrophotometer. Samples were heated from 15 °C → 90 °C and cooled from 90 °C → 20 °C at a rate of 1 °C min^−1^, and values were obtained from the maxima of the negative first derivative of the melting curve.

### Polyacrylamide gel experiments

Experiments were performed on 12% native polyacrylamide gels with 1× TBE buffer and 10 mM potassium chloride, using 1× TBE buffer with 10 mM potassium chloride as a running buffer. Gels were run on Bio-rad Mini-PROTEAN® gel kits with a Bio-rad PowerPac (highest voltage: 5000 V/500 mA/400 W). After electrophoresis, gels were stained with Diamond™ nucleic acid dye and visualised under UV with an AlphaImager HP gel imager from Alpha Innotech.

#### Gel electromobility shift assays

50 μl aqueous solutions of DNA (1 μM) and thrombin (1 μM) in KCl (10 mM), Tris·HCl buffer (10 mM, pH 7.5), and glycerol (3%) were prepared. 10 μl of each sample was loaded into wells and gels were run at 100 V for 1 hour.

#### DNA–acrylamide gel copolymerisation experiments

100 μl aqueous samples containing 1 μM DNA, 12% acrylamide, 1× TBE buffer, and 10 mM potassium chloride were prepared. 10% APS in water (1 μl) and TEMED (1 μl) were added and 40 μl of each sample was then immediately loaded into two adjacent wells of a gel (20 μl per well). A three well gap was left between unmodified and Acrylamide-dT modified DNA as the solutions were found to react into neighbouring wells. The solution-containing wells were then left to set (typically 5 minutes). Once set, 10 μl of 12% polyacrylamide gel solution was added to the same wells and left to set. The gels were then rinsed with HPLC water and run at 100 V for 1 hour.

## Conflicts of interest

There are no conflicts to declare.

## Supplementary Material

RA-009-C9RA07570D-s001
