# Supplementary material for: Acrylamide-dT: a polymerisable nucleoside for DNA incorporation
Source: RSC Adv. 2019 Oct 3;9(54):31511–6. doi: 10.1039/c9ra07570d (PMC9072585; doi:10.1039/c9ra07570d)
Supplement: RA-009-C9RA07570D-s001 [file RA-009-C9RA07570D-s001.pdf]

Electronic Supplementary Material (ESI) for RSC Advances.
This journal is © The Royal Society of Chemistry 2019

Supporting Information

Acrylamide-dT: A Polymerisable Nucleoside for DNA Incorporation

Francia Allabush[a,b], Paula M. Mendes[b] and James H. R. Tucker[a]

[a] School of Chemistry, University of Birmingham, Edgbaston, Birmingham, UK, B15 2TT
[b] School of Chemical Engineering, University of Birmingham, Edgbaston, Birmingham, UK, B15 2TT

Contents

1. NMR spectra of Acrylamide-dT and derivatives **S2**

2. Testing oligonucleotide deprotection conditions on Acrylamide-dT **S9**

3. Purification and characterisation of unmodified and Acrylamide-dT modified TBA **S13**

4. Gel electromobility shift assays of unmodified and Acrylamide-dT modified TBA with thrombin **S22**

1. NMR spectra of Acrylamide-dT and derivatives

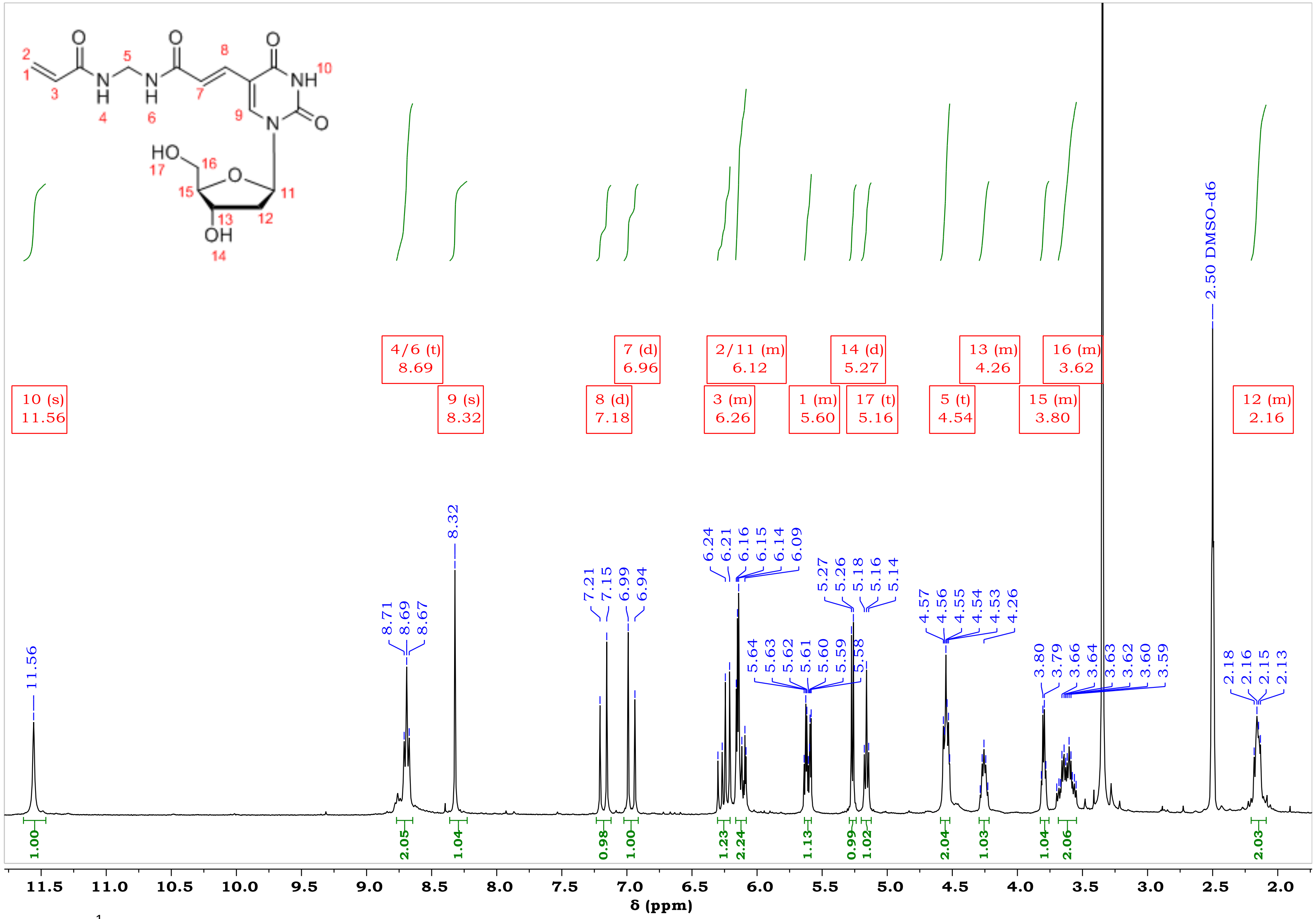

Figure S1- ^{1}H NMR spectrum of Acrylamide-dT in deuterated DMSO

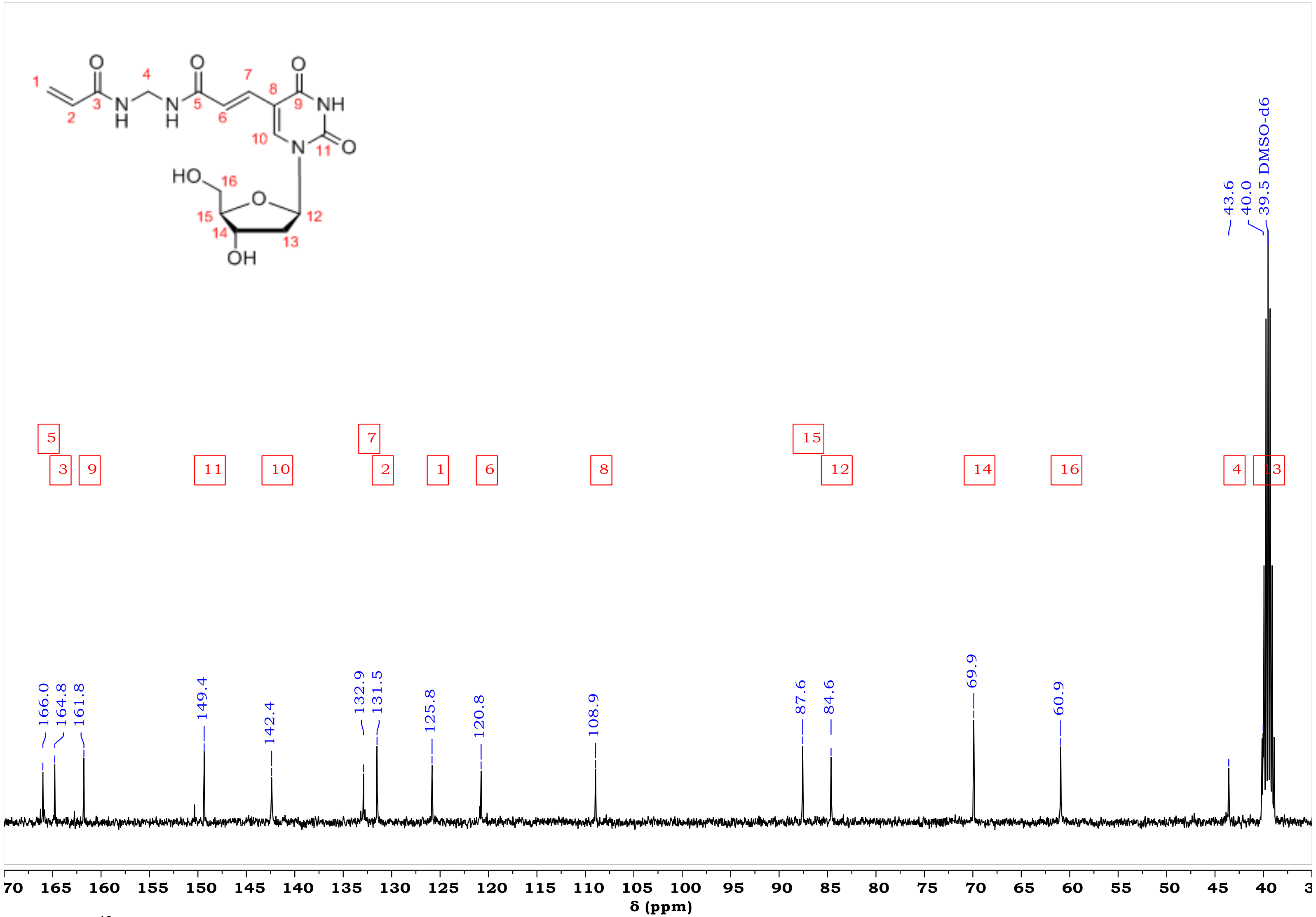

Figure S2- ^{13}C NMR spectrum of Acrylamide-dT in deuterated DMSO

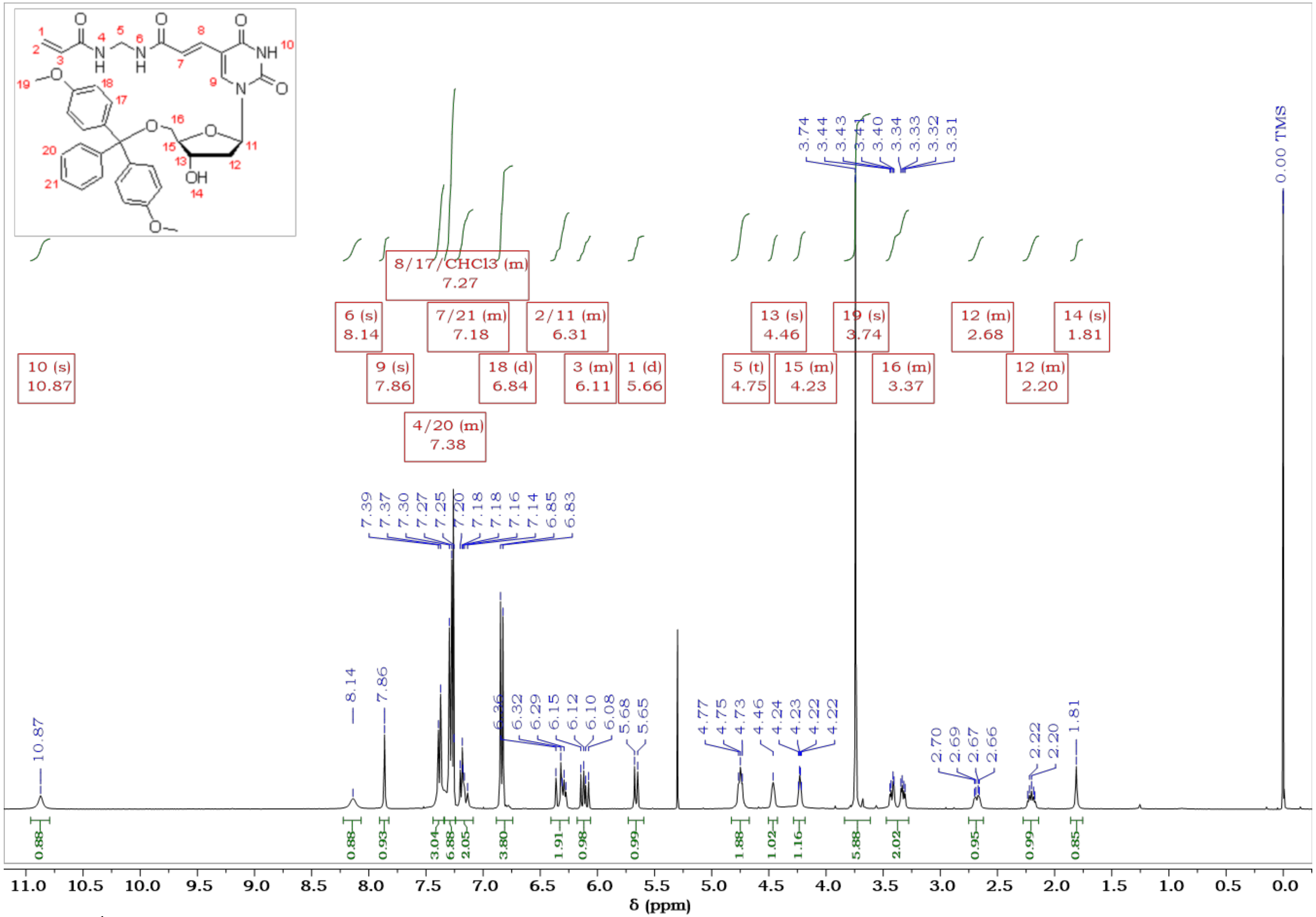

Figure S3- ^{1}H NMR spectrum of compound **3** in deuterated chloroform

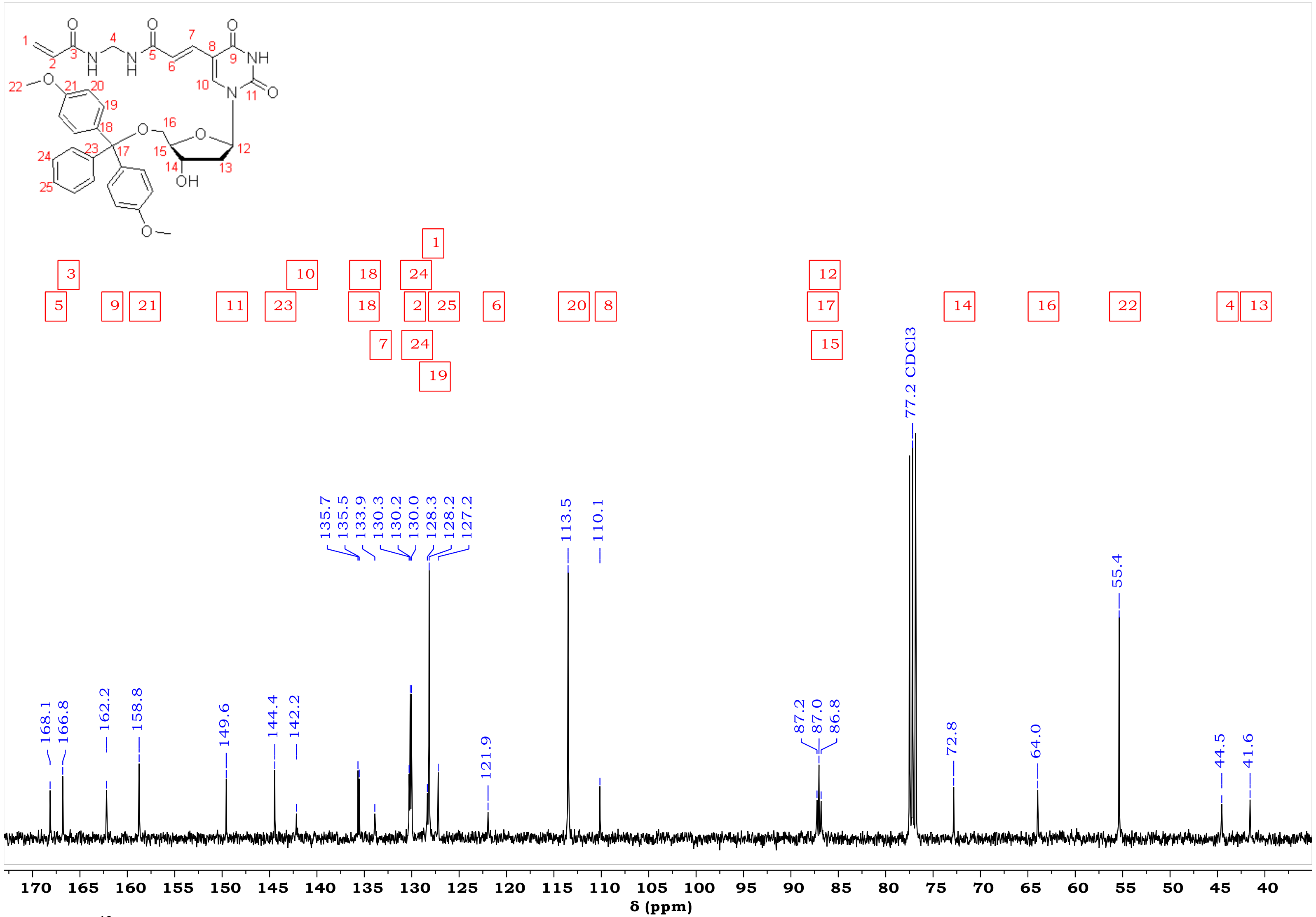

Figure S4- ^{13}C NMR spectrum of compound **3** in deuterated chloroform

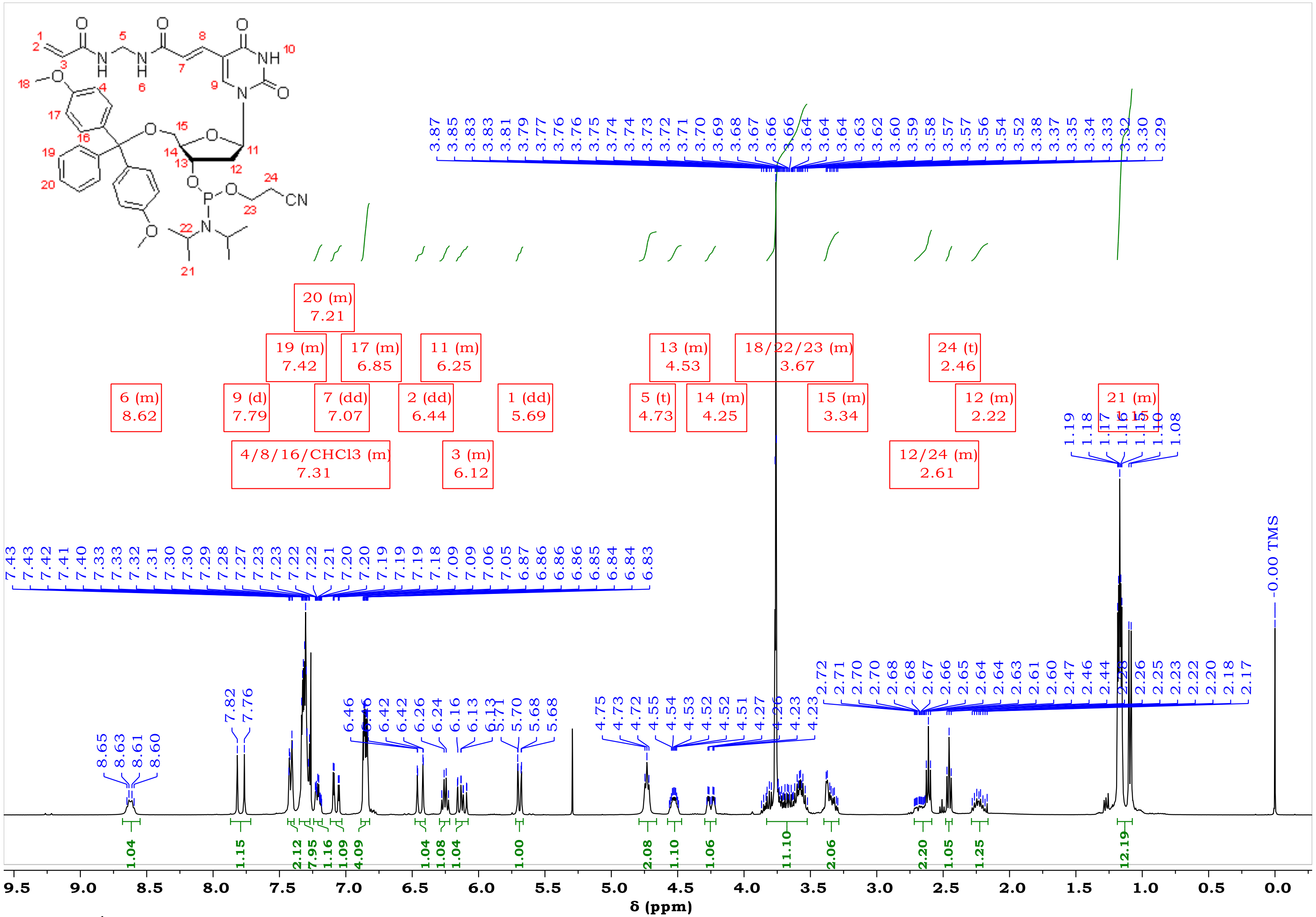

Figure S5- ^{1}H NMR spectrum of Acrylamide-dT-CE phosphoramidite in deuterated chloroform

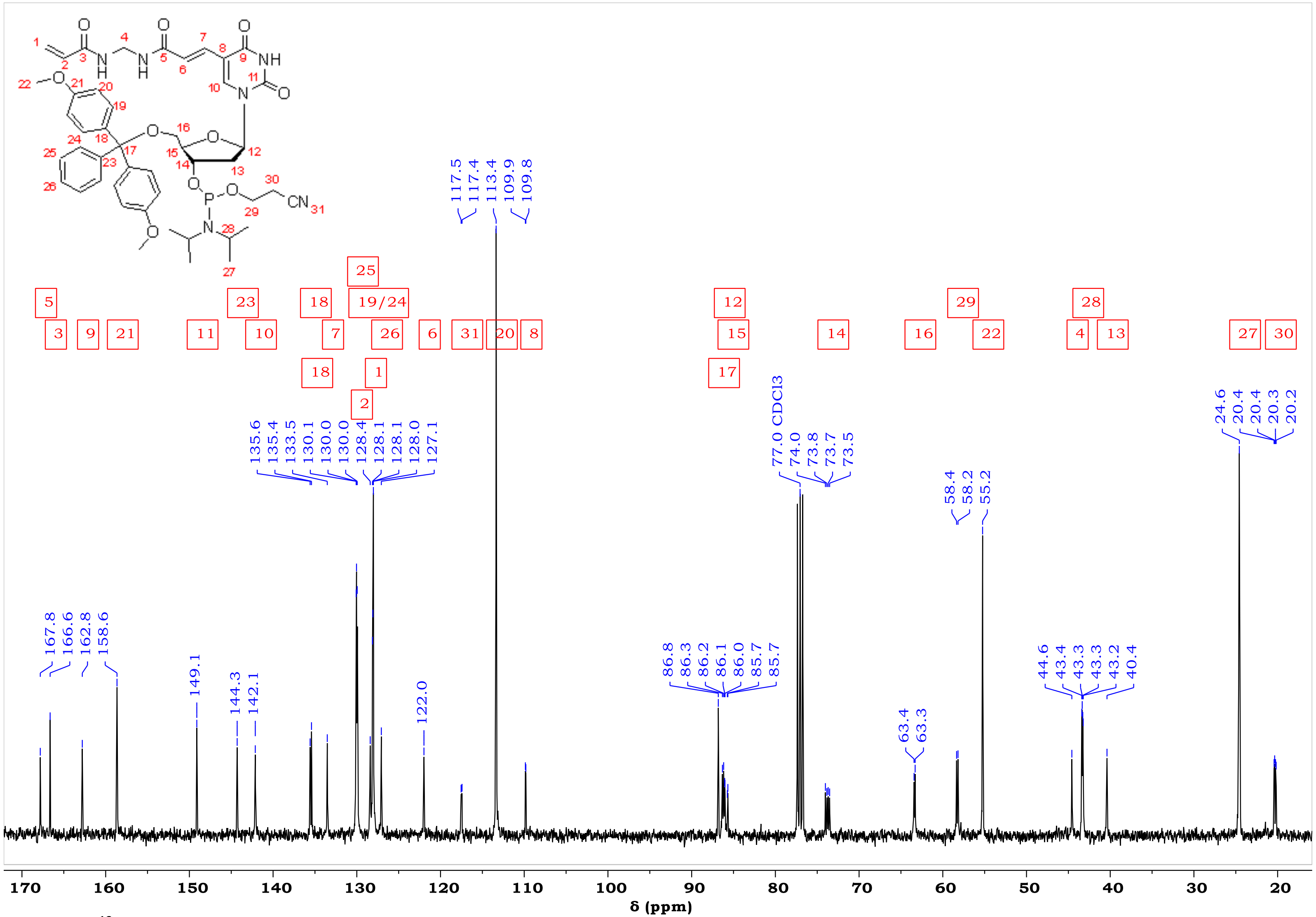

Figure S6- ^{13}C NMR spectrum of Acrylamide-dT-CE phosphoramidite in deuterated chloroform

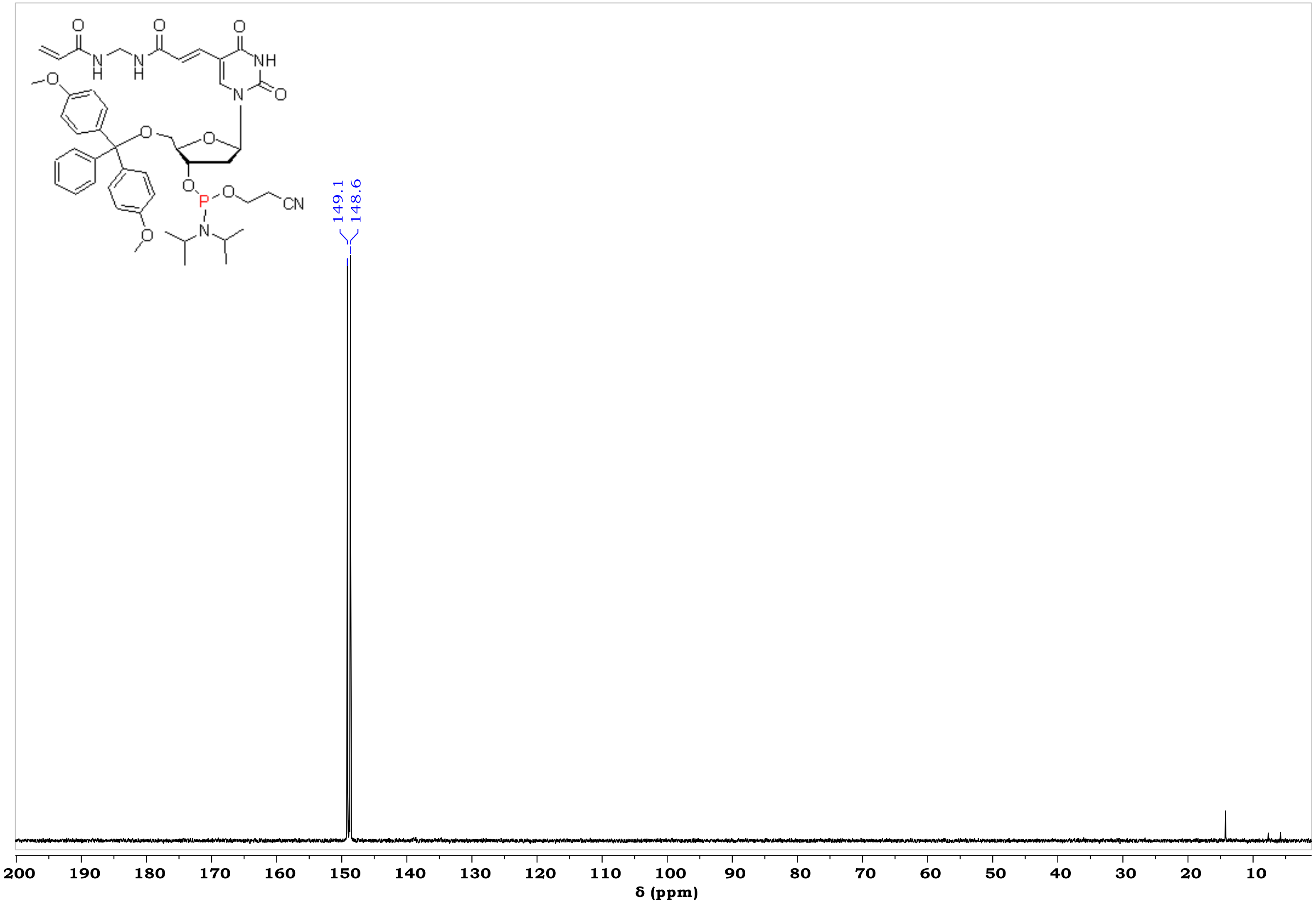

Figure S7- ^{31}P NMR spectrum of Acrylamide-dT-CE phosphoramidite in deuterated chloroform

2. Testing oligonucleotide deprotection conditions on Acrylamide-dT

For standard deprotection conditions: Acrylamide-dT (10mg) was added to a 1ml solution of aqueous ammonia (30%). The resulting suspension was sealed, shaken, and heated to 60°C for 6 hours. The solvent was then evaporated, and the crude dissolved in deuterated DMSO and analysed by ^{1}H, ^{13}C, and 2D NMR. The solid crude was also analysed by mass spectrometry.

For ultramild deprotection conditions: Acrylamide-dT (10mg) was added to a 1ml freshly prepared solution of potassium carbonate in methanol (0.05M). The resulting suspension was sealed, shaken, and left standing at room temperature for 12 hours. The solvent was then evaporated, and the crude dissolved in deuterated DMSO, and analysed by ^{1}H NMR.

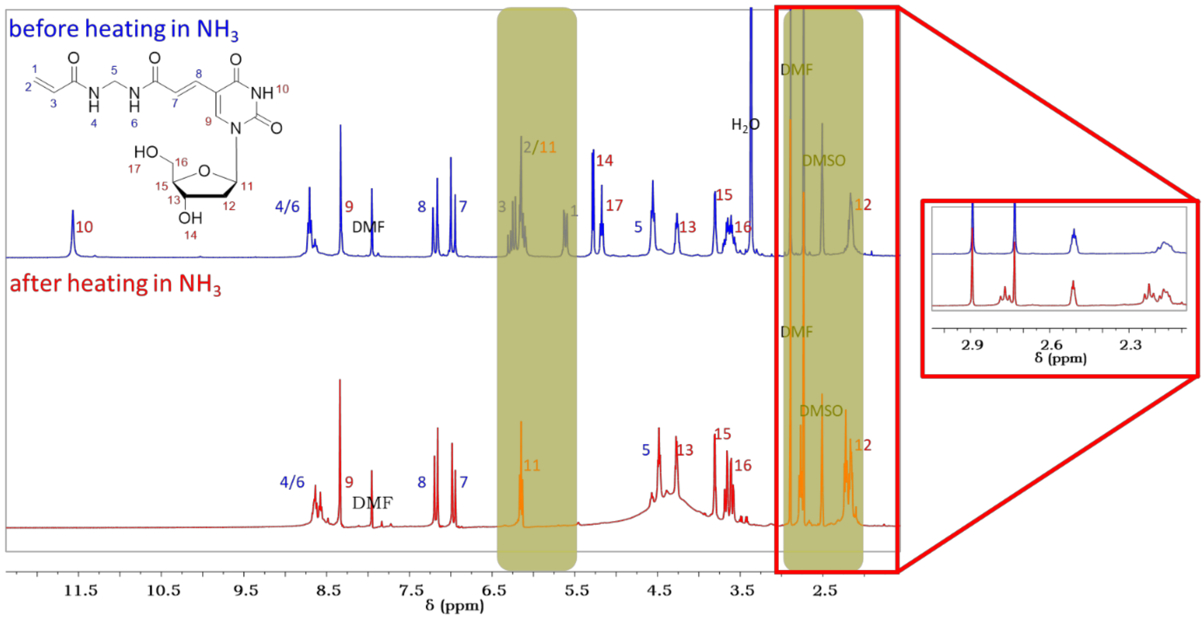

Figure S8- ^{1}H NMR spectra of Acrylamide-dT in deuterated DMSO before and after heating in aqueous ammonia (highlighted sections denote significant changes to the spectra).

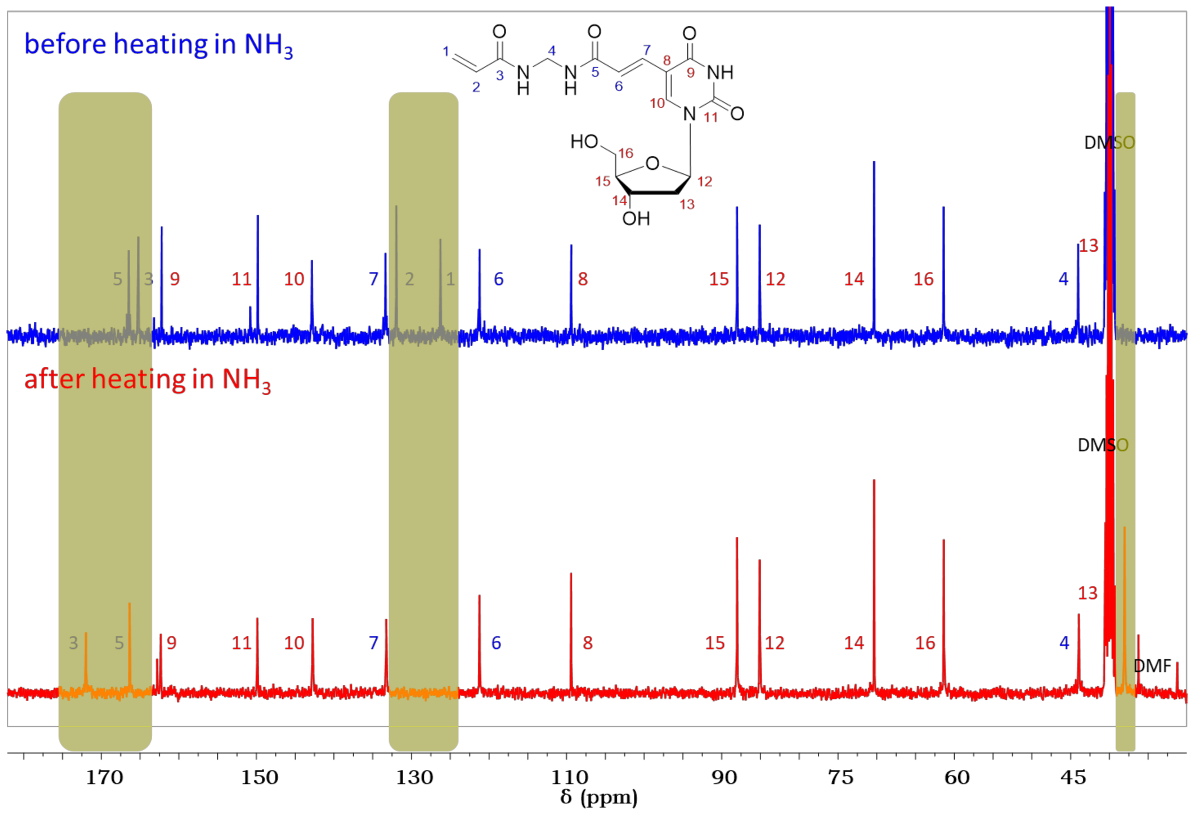

Figure S9- ^{13}C NMR spectra of Acrylamide-dT in deuterated DMSO before and after heating in aqueous ammonia (highlighted sections denote significant changes to the spectra).

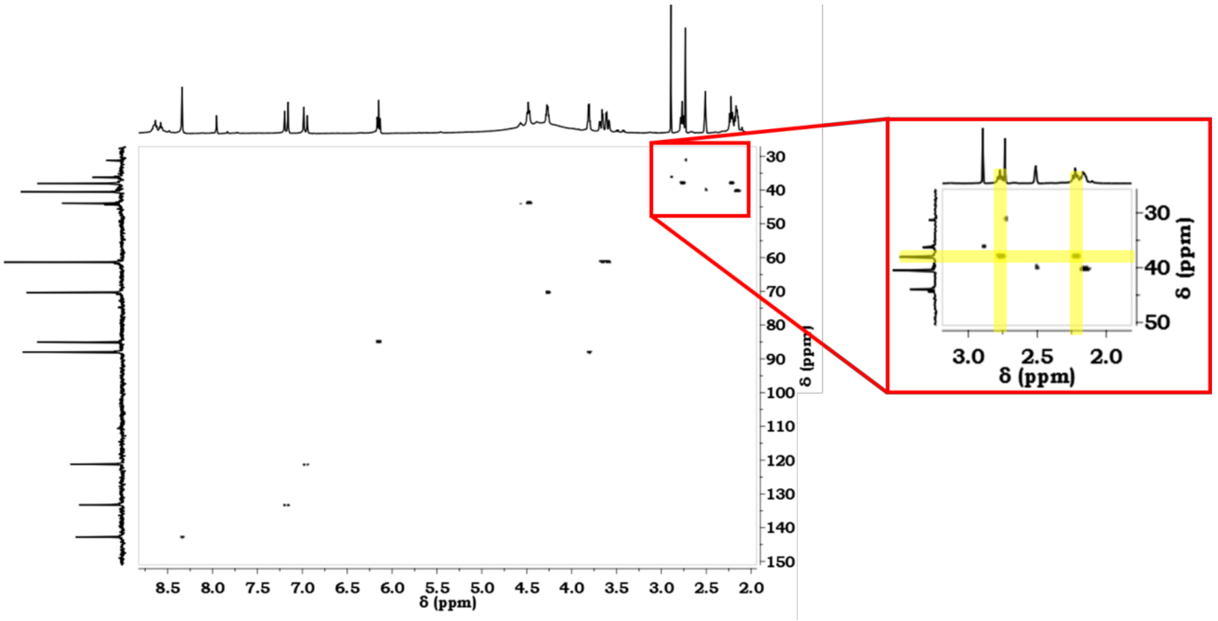

Figure S10- HSQC NMR spectrum of Acrylamide-dT in deuterated DMSO after heating with aqueous ammonia (highlighted sections denote significant changes to the spectra).

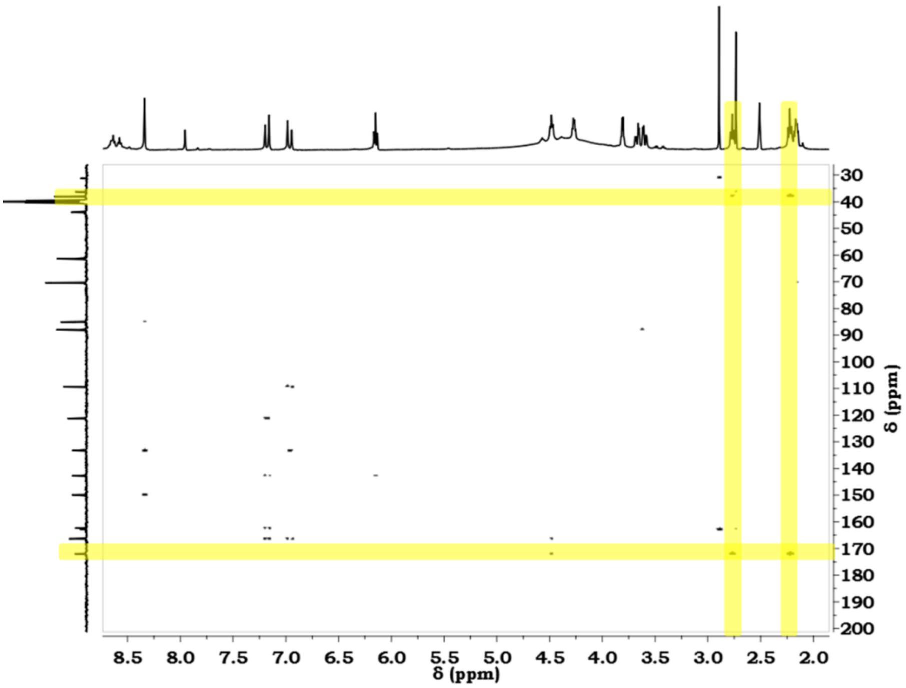

Figure S11- HMBC NMR spectrum of Acrylamide-dT in deuterated DMSO after heating with aqueous ammonia (highlighted sections denote significant changes to the spectra).

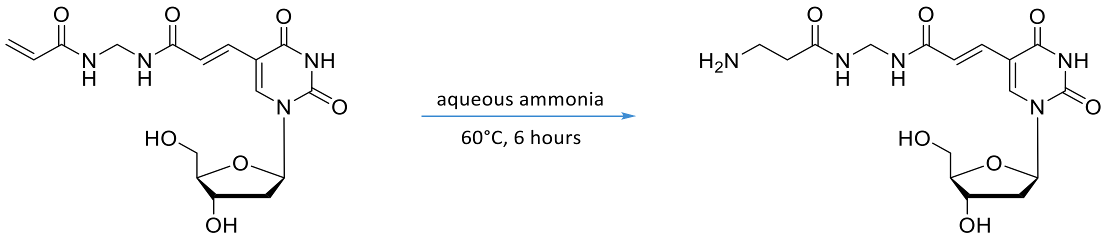

Scheme S1- Reaction of Acrylamide-dT with hot aqueous ammonia

The changes to the NMR spectra (Figures S8 – S11) are consisent with NH_3 reacting with the acrylamide group of Acrylamide-dT as shown above. In contrast, the treatment of Acrylamide-dT with methanolic potassium carbonate resulted in no changes to the spectrum, as shown below in Figure S12.

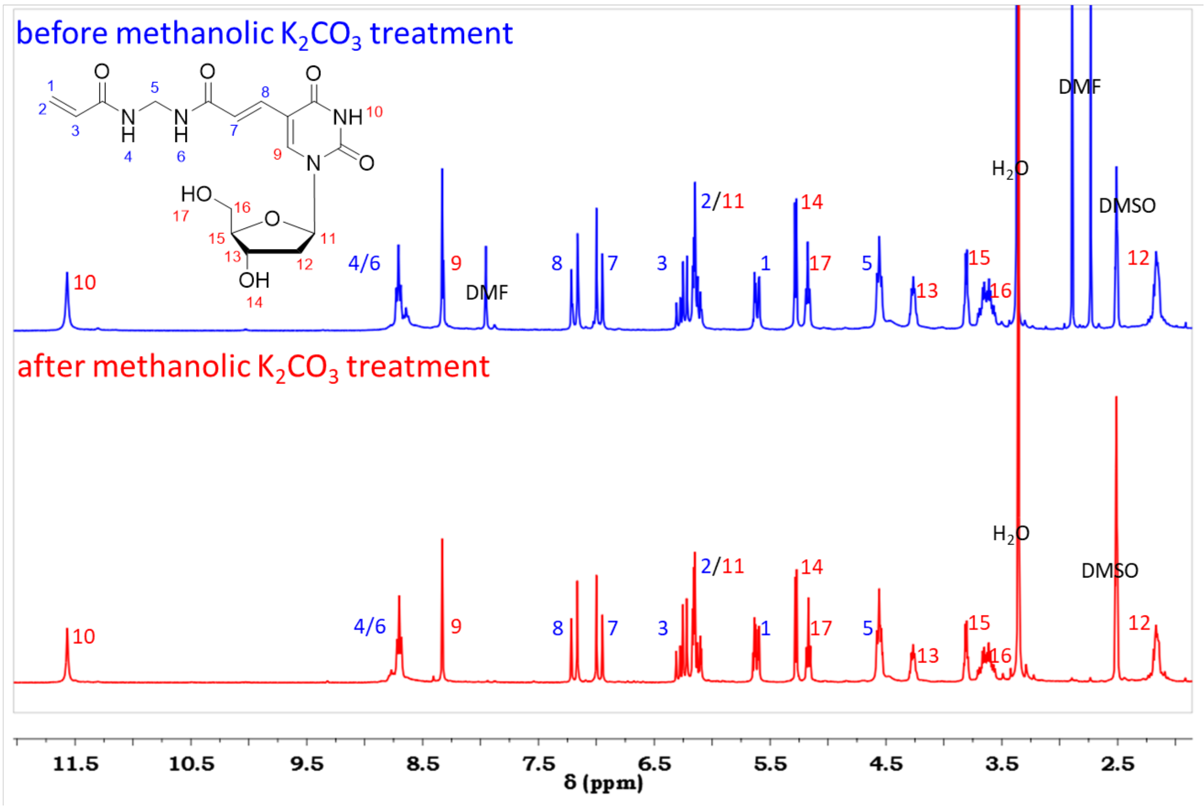

Figure S12- ^{1}H NMR spectra of Acrylamide-dT in deuterated DMSO before and after treatment with potassium carbonate in methanol.

3. Purification and characterisation of unmodified and Acrylamide-dT modified TBA

Semi preparative HPLC purification was performed on an Agilent Technologies 1260 Infinity system using a Phenomenex Clarity 5 µm Oligo-RP LC 250 x 10 mm column. 1 ml of sample was injected with a run time of 45 minutes for each sample, at a flow rate of 3 ml/min. The column was heated to 60 °C prior to sample injection. The UV/vis absorbance of each run was monitored at 260 nm. The solvent gradients used are listed in the table below. Collected fractions were evaporated to dryness, diluted to 1 ml in Milli-Q water, and desalted using a NAP-10 column (GE Healthcare), whilst eluting to 1.5 ml.

Purity of oligonucleotides was determined by analytical HPLC using a Phenomenex Clarity 5 µm Oligo RP LC 250 x 4.6 mm column on either a Shimadzu HPLC or an Agilent Technologies 1260 Infinity system. The column was heated to 60 °C prior to sample injection. 20 µl of sample was injected with a run time of 45 minutes for each sample, at a flow rate of 1 ml/min. Solvent gradients used were identical to semi preparative HPLC. The UV/vis absorbance of each run was monitored at 260 nm. Samples showing >95% purity by analytical HPLC were deemed sufficiently pure for use in experiments. Samples showing <95% purity were repurified by semi preparative HPLC.

The characterisation of pure oligonucleotide samples was performed by negative mode ESMS. Sample concentrations were determined by optical density at 260 nm using a BioSpec-nano micro-volume UV-Vis spectrophotometer (nanodrop) from Shimadzu and the Beer Lambert law, with extinction coefficients obtained from Integrated DNA Technologies' OligoAnalyzer.

Time/mins	0.1M TEAA aq./%	Acetonitrile/%
0	95	5
30	82	18
30.1	0	100
40	0	100
40.1	95	5
45	95	5

Oligo name	Oligo sequence (X=Acrylamide-dT)	HPLC purity (%)	Calculated mass (*m/z*)	Observed mass (*m/z*)
**unmodified TBA**	5'-GGTTGGTGTGGTTGG-3'	97	4726	4726
**Acryl-endT**	5'-**X**GGTTGGTGTGGTTGG-3'	97	5168	5168
**Acryl-T7/T9**	5'-GGTTGG**X**G**X**GGTTGG-3'	96	5002	5002
**Acryl-endT/T7/T9**	5'-**X**GGTTGG**X**G**X**GGTTGG-3'	99	5442	5481

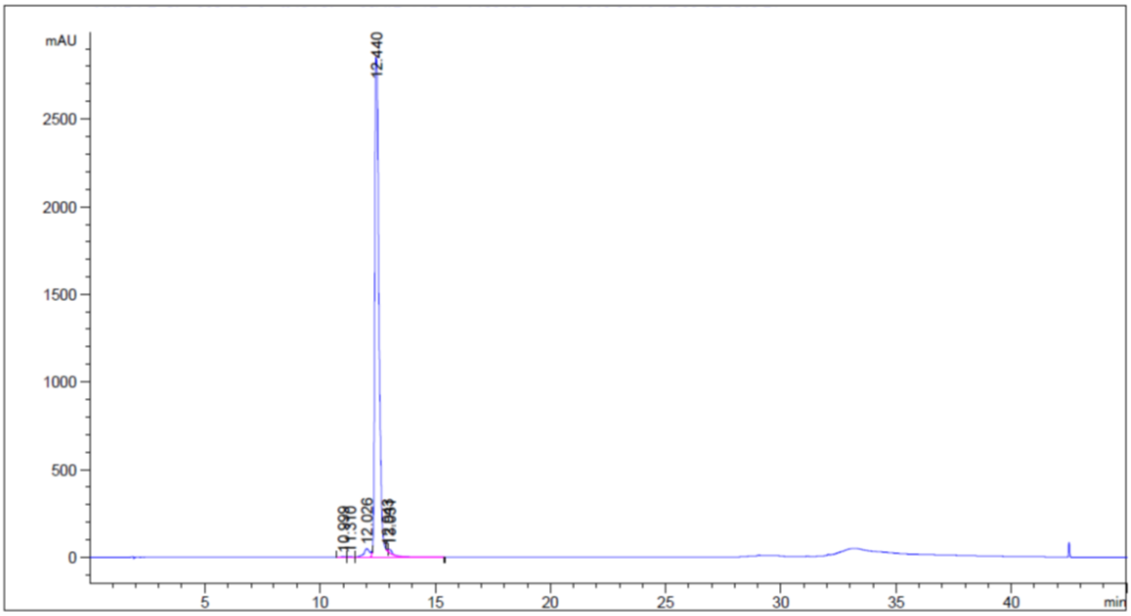


```
=====================================================================
                          Area Percent Report
=====================================================================

Sorted By             :      Signal
Multiplier            :      1.0000
Dilution              :      1.0000
Do not use Multiplier & Dilution Factor with ISTDs


Signal 1: MWD1 F, Sig=260,4 Ref=off

Peak RetTime Type  Width     Area      Height     Area
  #   [min]        [min]   [mAU*s]     [mAU]       %
----|-------|----|-------|----------|----------|--------|
   1  10.999 BV    0.1414   39.91587    4.17932   0.1045
   2  11.310 VB    0.1507   35.89089    3.58824   0.0940
   3  12.026 BV E  0.2617  871.17682   47.55590   2.2805
   4  12.440 VV R  0.1992 3.69481e4  2850.73071  96.7195
   5  12.943 VV E  0.0721   51.30489    9.95322   0.1343
   6  13.031 VB E  0.1729  254.88455   20.52154   0.6672
```

Figure S13- Analytical HPLC chromatogram of unmodified TBA

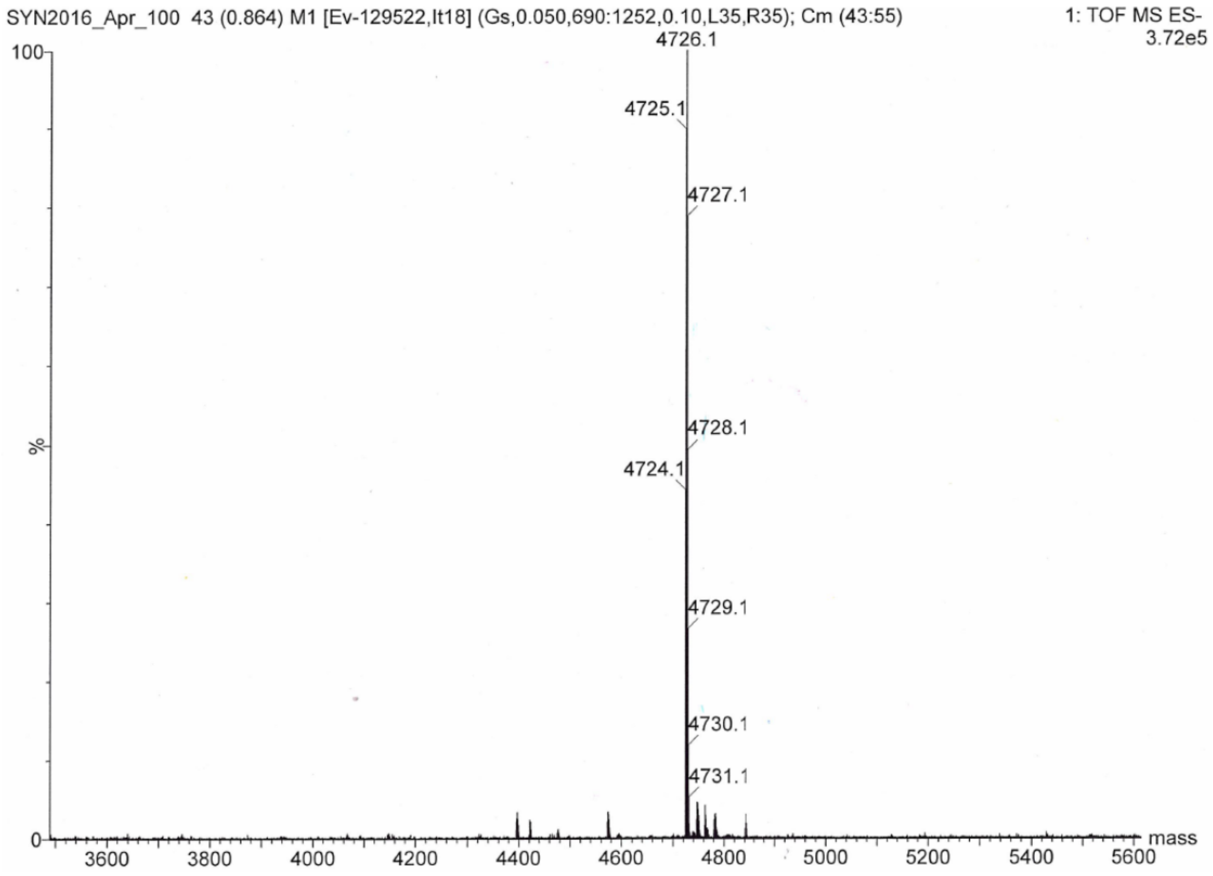

Figure S14- Mass spectrum of unmodified TBA

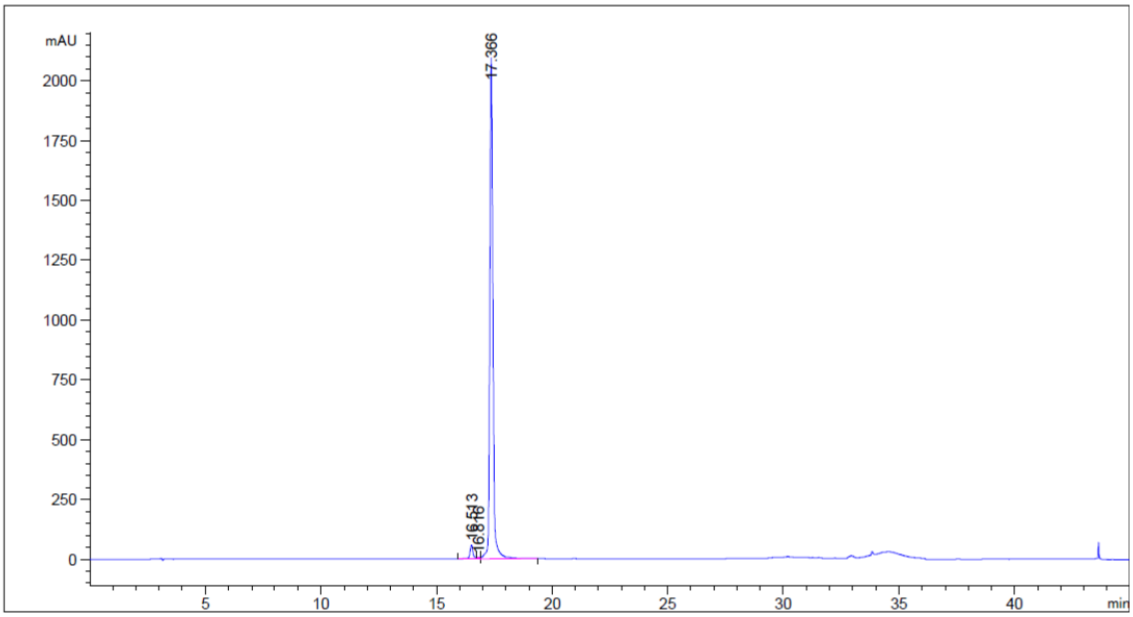


```
=====================================================================
                         Area Percent Report
=====================================================================

Sorted By             :      Signal
Multiplier            :      1.0000
Dilution              :      1.0000
Do not use Multiplier & Dilution Factor with ISTDs


Signal 1: MWD1 F, Sig=260,4 Ref=off

Peak RetTime Type  Width     Area      Height     Area
  #   [min]        [min]   [mAU*s]     [mAU]        %
----|-------|----|-------|----------|----------|--------|
   1  16.513 BV R  0.1435  550.02069   56.54726   2.7464
   2  16.816 VV E  0.1036   12.60930    1.75927   0.0630
   3  17.366 VV R  0.1404 1.94646e4  2095.81494  97.1907
```

Figure S15- Analytical HPLC chromatogram of Acryl-endT

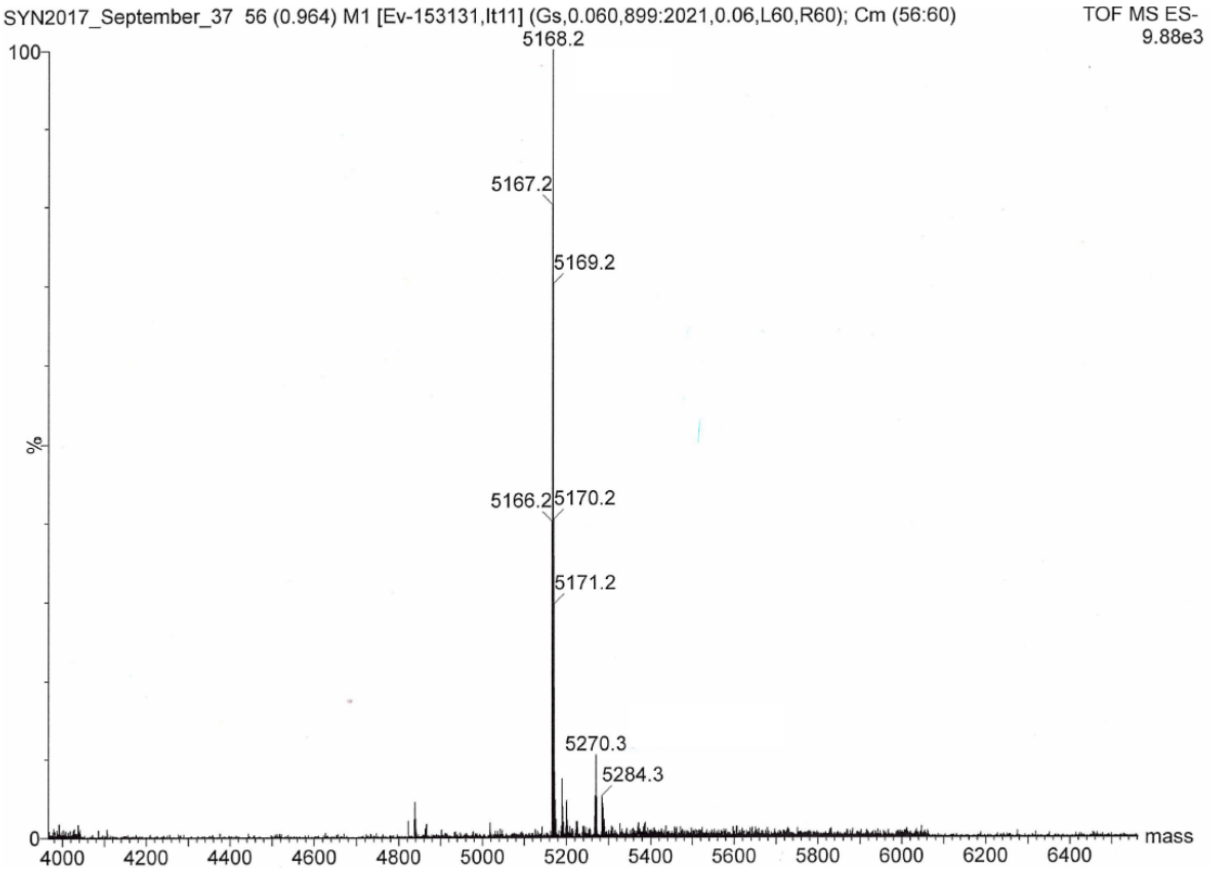

Figure S16- Mass spectrum of Acryl-endT

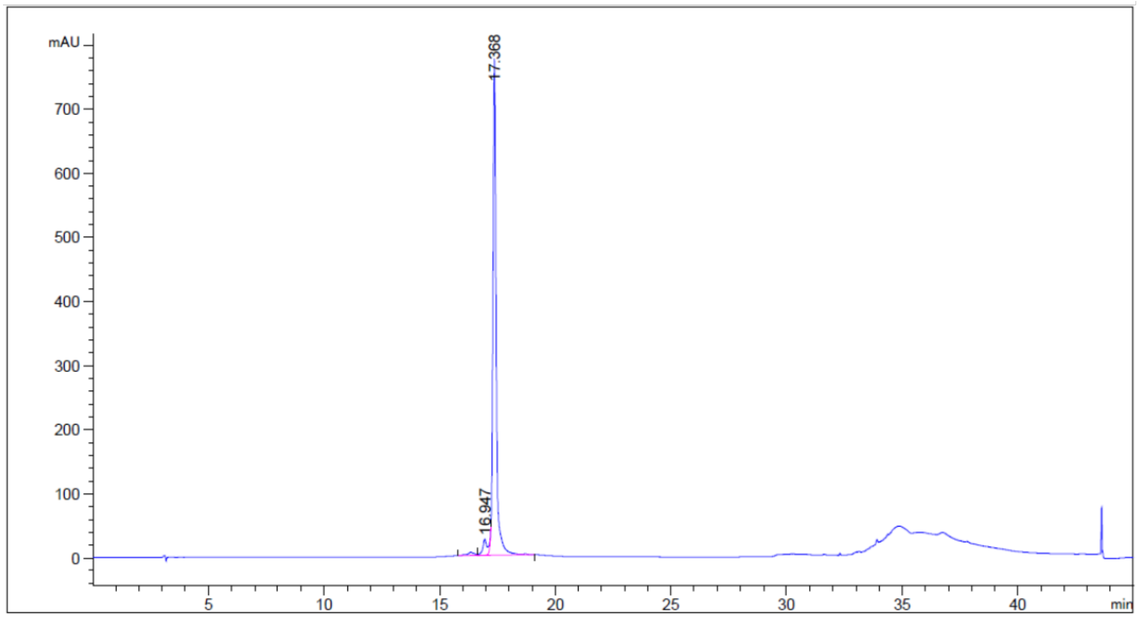


```
=====================================================================
                        Area Percent Report
=====================================================================

Sorted By             :      Signal
Multiplier            :      1.0000
Dilution              :      1.0000
Do not use Multiplier & Dilution Factor with ISTDs


Signal 1: MWD1 F, Sig=260,4 Ref=off

Peak RetTime Type  Width     Area      Height     Area
  #   [min]        [min]   [mAU*s]     [mAU]       %
----|-------|----|-------|----------|----------|--------|
   1  16.947 VV E  0.2009  366.27063   25.60049   4.4879
   2  17.368 VV R  0.1455 7795.06494  775.17017  95.5121
```

Figure S17- Analytical HPLC chromatogram of Acryl-T7/T9

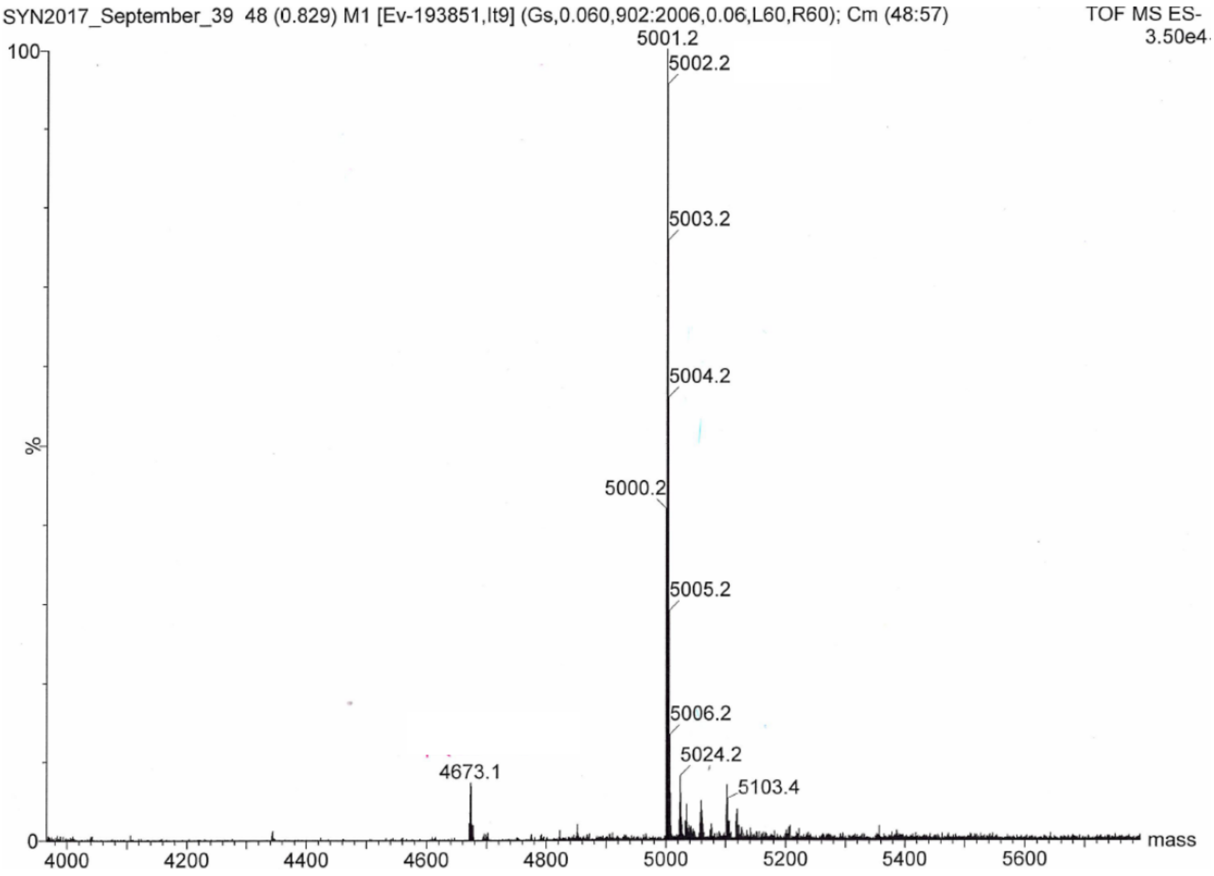

Figure S18- Mass spectrum of Acryl-T7/T9

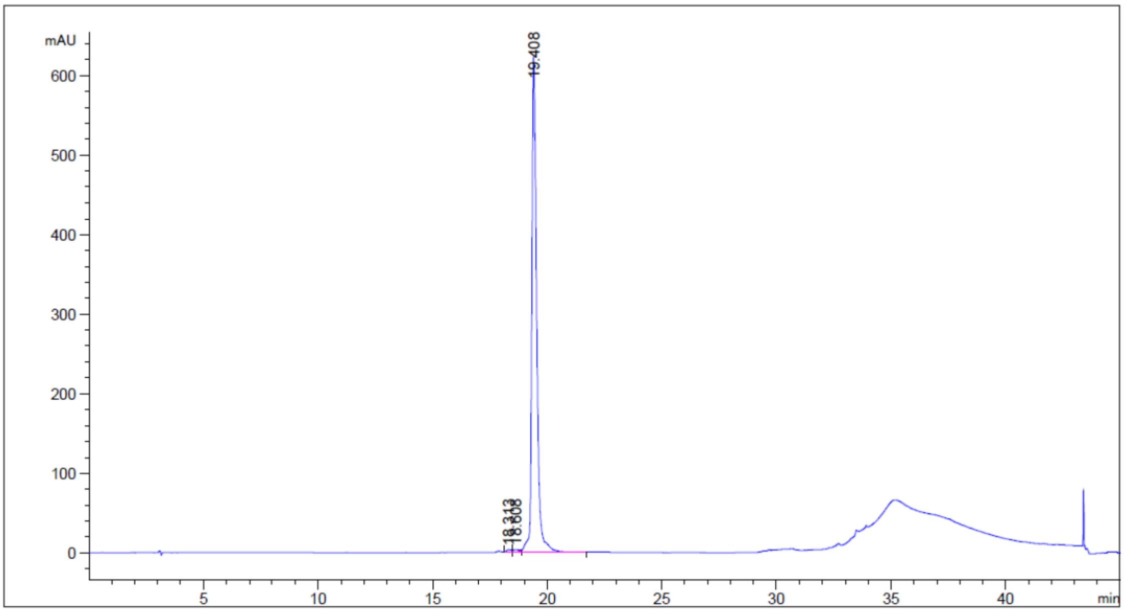


```
=====================================================================
                         Area Percent Report
=====================================================================

Sorted By             :      Signal
Multiplier            :      1.0000
Dilution              :      1.0000
Do not use Multiplier & Dilution Factor with ISTDs


Signal 1: MWD1 F, Sig=260,4 Ref=off

Peak RetTime Type  Width     Area      Height     Area
  #   [min]        [min]   [mAU*s]     [mAU]        %
----|-------|----|-------|----------|----------|--------|
   1  18.313 BV E  0.2067   42.35224    3.11375   0.4540
   2  18.608 VV E  0.2198   53.16344    3.30259   0.5699
   3  19.408 VB R  0.2290 9232.51855  622.73004  98.9760
```

Figure S19- Analytical HPLC chromatogram of Acryl-endT/T7/T9

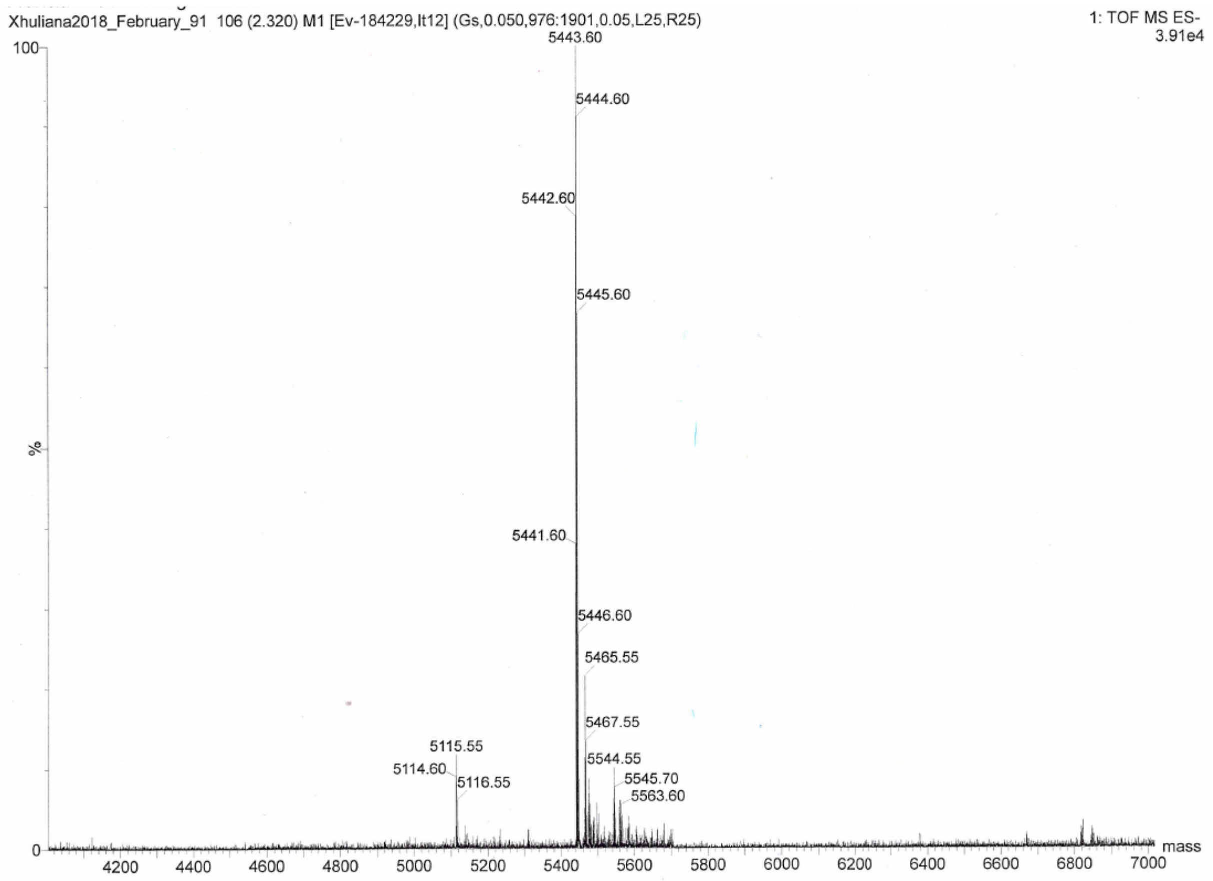

Figure S20- Mass spectrum of Acryl-endT/T7/T9

4. Gel electromobility shift assays of unmodified and Acrylamide-dT modified TBA with thrombin

Experiments were performed on 12% native polyacrylamide gels with 1 x TBE buffer and 10mM potassium chloride, using 1x TBE buffer with 10mM potassium chloride as a running buffer. Gels were run on Bio-rad Mini-PROTEAN® gel kits with a Bio-rad PowerPac (highest voltage: 5000V/500mA/400W). After electrophoresis, gels were stained with Diamond™ nucleic acid dye and visualised under UV with an AlphaImager HP gel imager from Alpha Innotech.

Gel electromobility shift assays: 50µl aqueous solutions of DNA (1 µM) and thrombin (1 µM) in KCl (10 mM), tris.HCl buffer (10 mM, pH 7.5), and glycerol (3%) were prepared. 10µl of each sample was loaded into wells and gels were run at 100V for 1 hour.

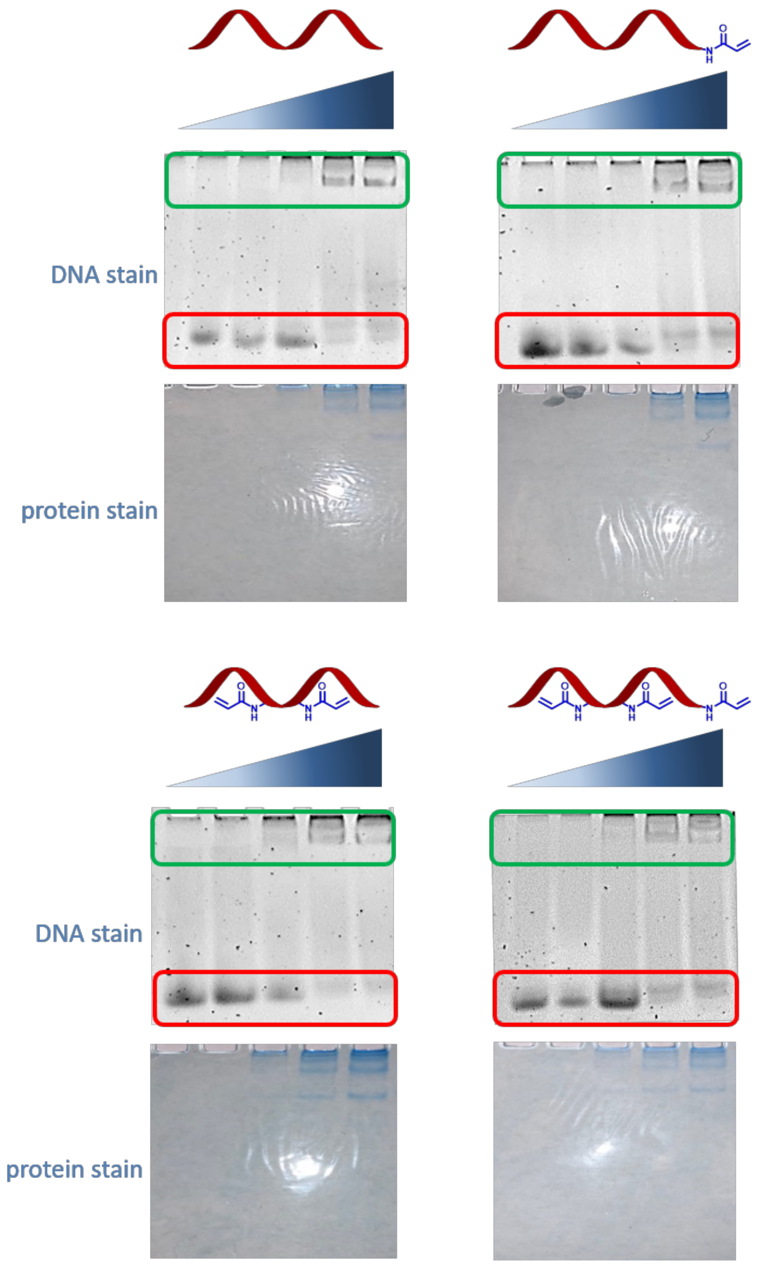

Figure S21- Gel EMSA of unmodified and Acrylamide-dT modified TBA (0.5µM) with increasing concentrations of thrombin (0→1 µM). Positions of TBA bands are highlighted in red and TBA-thrombin complex bands are highlighted in green. TBA:thrombin ratios used in each lane are as follows: lane 1- 1:0, lane 2- 1:0.02, lane 3- 1:0.2, lane 4- 1:1, lane 5- 1:2.
